# Ancient grain flour consumption as a novel therapeutic approach for irritable bowel syndrome

**DOI:** 10.1007/s00394-025-03859-8

**Published:** 2025-12-19

**Authors:** Samantha Maurotti, Yvelise Ferro, Elisa Mazza, Luca Tirinato, Rosario Mare, Miriam Frosina, Angela Mirarchi, Francesca Rita Noto, Patrizia Doldo, Carmen Colaci, Nicola d’Avanzo, Angelo Galluccio, Simona Greco, Arturo Pujia, Tiziana Montalcini

**Affiliations:** 1 Department of Clinical and Experimental Medicine, University “Magna Græcia” of Catanzaro, 88100 Catanzaro, Italy; 2 Department of Medical and Surgical Sciences, University “Magna Græcia” of Catanzaro, 88100 Catanzaro, Italy; 3 Department of Health Sciences, University “Magna Græcia” of Catanzaro, 88100 Catanzaro, Italy; 4 Research Center “ProHealth Translational Hub”, University “Magna Græcia” of Catanzaro, 88100 Catanzaro, Italy; 5 Research Center for the Prevention and Treatment of Metabolic Diseases, University “Magna Græcia”, 88100 Catanzaro, Italy

**Keywords:** Irritable bowel syndrome, Ancient grains, *Triticum monococcum*, Jermano wheat, Functional pasta, Sustainable food

## Abstract

**Background:**

Climate change has revived interest in ancient grains for their sustainability and nutritional value. Less processed and more resilient than modern cereals, they have a lower environmental impact. Grains like *Triticum monococcum* and Jermano wheat may benefit IBS patients due to their better digestibility. This study explores their use in functional pasta to manage IBS symptoms.

**Methods:**

Forty-two IBS patients followed a 4-week low-FODMAP diet with either traditional gluten-free pasta or functional pasta (FP) made from emmer and rye flour. IBS symptoms were assessed using the IBS-SSS questionnaire at baseline and after intervention. In vitro, functional pasta extract (FPE) was tested on TNF-α-induced Caco-2 and T84 cells to evaluate its effects on inflammatory and oxidative stress markers.

**Results:**

Patients consuming FP showed a significant reduction in IBS-SSS scores compared to the control group (− 153 ± 124 vs. − 83 ± 85, *p* = 0.044). Additionally, 83% of those on FP reported a clinically significant reduction in symptom severity (IBS-SSS score ≥ 100) compared to 35% in the control group (*p* = 0.004). In Caco-2 and T84 cells, FPE has been shown to downregulate protein expression of the pErk1/2 and NF-κB signaling pathways, thereby reducing inflammatory oxidative stress markers.

**Conclusion:**

These findings suggest that consuming functional pasta made with ancient grains could enhance gut health and alleviate symptoms of IBS by reducing inflammation and oxidative stress. This innovative dietary approach offers a promising natural alternative for improving the quality of life in IBS patients.

**Trial registration:**

Trial registration ISRCTN, ISRCTN12170245 Registered 13 june 2025—Retrospectively Registered, https://doi.org/10.1186/ISRCTN12170245.

**Graphical abstract:**

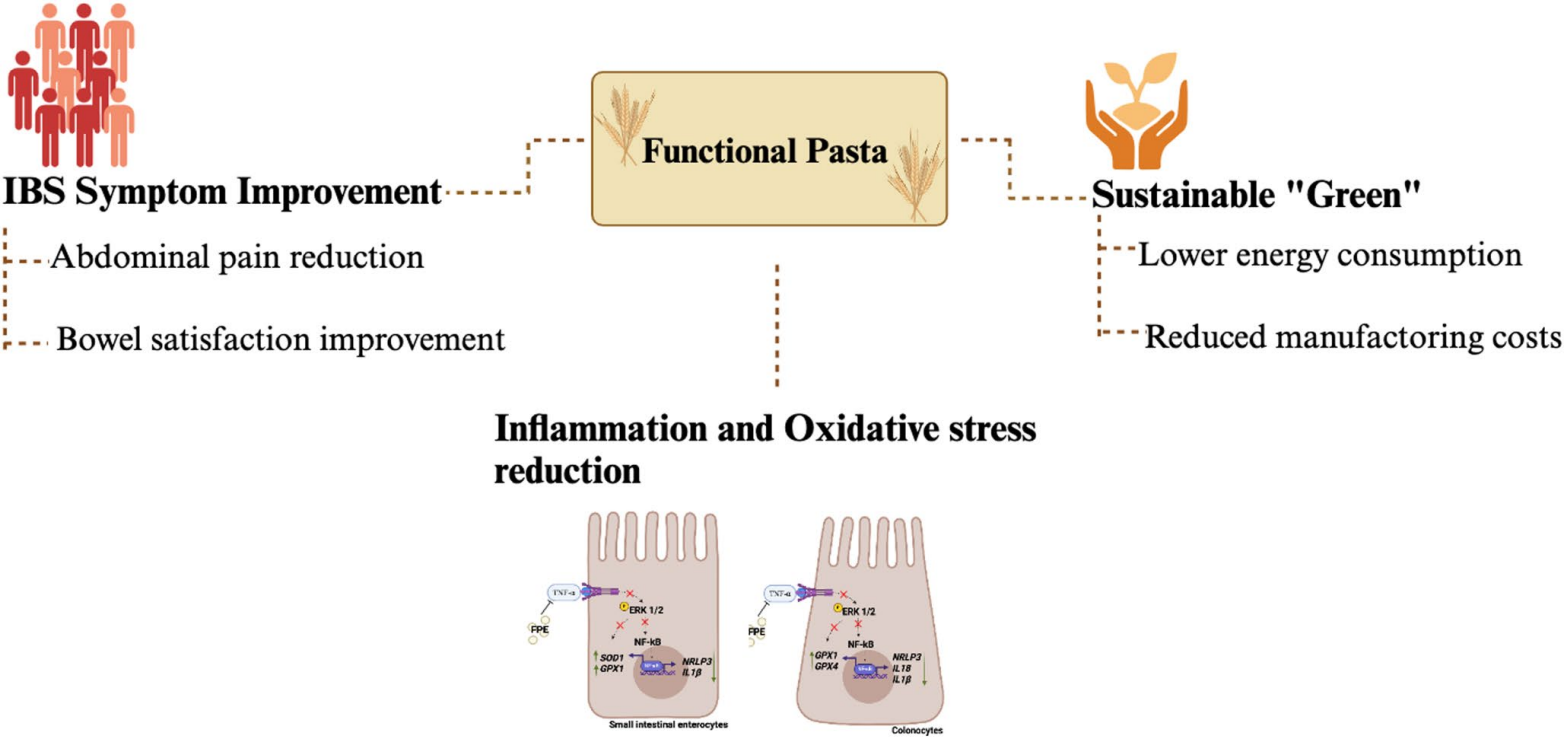

**Supplementary Information:**

The online version contains supplementary material available at 10.1007/s00394-025-03859-8.

## Introduction

In the face of climate change and its impact on global food systems, there is growing interest in finding sustainable agricultural solutions. One promising approach lies in the use of ancient cereals, which offer a viable alternative to conventional crops [1]. Cereals are classified as "modern" or "ancient" based on their genetic background. Unlike modern cereals, ancient ones are underutilized crops not selected through breeding programs [2]. Reintroducing these cereals into agriculture offers multiple benefits, including improved health outcomes, reduced environmental impact, and increased food availability [3–5]. Studies have also highlighted their nutritional richness, unique physicochemical properties, diverse food applications, and contribution to sustainability and dietary diversity, underscoring their potential to address global food challenges [1, 6, 7].

Since early history, ancient grains have served as a fundamental food source. However, the selection of high-yield domestic species with enhanced techno-functional properties led to a drastic decline in their cultivation, favoring a few dominant crops such as wheat, rice, maize, and barley [3]. Recently, ancient cereals have regained attention due to their environmental sustainability, as they require less irrigation, pesticides, and fertilizers, resulting in a lower carbon footprint [8, 9]. Their resilience to harsh growing conditions makes them ideal for climate-smart agriculture. Additionally, they are rich in nutrients and bioactive compounds, offering significant health benefits [1, 5, 9].

Ancient grains have shown benefits in addressing health issues, including cardiovascular diseases, type 2 diabetes, cancer, weight management, and digestive disorders [8, 10, 11]. Moreover, they are rich in nutrients and bioactive compounds, offering significant health benefits, including potential lipid modulation similar to other functional foods like nuts [12]. Their consumption has also been linked to improved gastrointestinal symptoms, such as Irritable Bowel Syndrome (IBS), and reduced inflammation across various populations [13, 14].

IBS is a prevalent functional bowel disorder characterized by recurrent abdominal pain linked to defecation or alterations in bowel habits, manifesting as constipation, diarrhea, or both, often accompanied by abdominal bloating or distension [15]. Diagnostic criteria require symptoms to persist for at least six months before diagnosis, with active symptoms within the last three months [15]. The disorder affects approximately 11.2% of the global population, predominantly women and individuals under 50 years of age [16]. IBS significantly diminishes quality of life, incurs high healthcare costs, and contributes to increased work absenteeism [17, 18].

The pathophysiology of IBS involves complex interactions between gut microbiota disturbances, low-grade inflammation, psychological factors, and dietary components [15, 19, 20]. Dietary management, particularly through modification of meal patterns and specific dietary elements, plays a central role in symptom management. The National Institute for Health and Care Excellence (NICE) guidelines recommend dietary adjustments such as regular small meals, reduced intake of gas-producing vegetables, limited fiber, and minimized consumption of coffee and tea [21]. Furthermore, the low FODMAP diet, restricting fermentable carbohydrates, has gained acceptance for its effectiveness in alleviating symptoms by reducing osmotic stress and fermentation-induced distension in the gut [22] but yet nearly half of IBS patients report inadequate relief from such interventions. FODMAPs are widespread in food products, particularly in staple items like bread and pasta [23], which contributes to low dietary adherence. However, nearly 50% of IBS patients do not find symptom relief following either NICE recommendations or a low FODMAP diet [24]. Additionally, emerging evidence suggests that non-celiac gluten sensitivity might exacerbate IBS symptoms, further complicating dietary management. Gluten, a protein complex found in many grains, has been implicated in increased gastrointestinal symptoms among IBS patients, attributed to its incomplete digestion by proteases, which can trigger an innate immune response [25–28]. Recent studies suggests that gluten can disrupt the intestinal barrier, affect microbiota composition, and enhance intestinal motility, particularly in IBS-D (diarrhea-predominant) patients [29]. Most ancient grains can serve as a healthy, highly nutritious alternative to modern cereals like wheat, rice, and maize. They cause fewer allergic reactions and are better tolerated in FODMAP diets compared to conventional grains [11, 30].

Research suggests that alternative grains may have a positive impact on IBS [29, 31]. Triticum monococcum, commonly known as einkorn wheat, is a member of the grass family grown in Calabria. It contains approximately 7% gluten and is suitable for bread and pasta making [32]. In vitro studies have shown that the gluten protein in emmer wheat is broken down during digestion, unlike the gluten in common wheat [33]. This makes einkorn gluten more fragile and digestible, and as a result, it is better tolerated by those with sensitivities to traditional wheat. *Jermano wheat*, also known as Calabrian rye, widely used in Southern Italy until the 1950s, has potential beneficial effects on IBS [34]. While rye is typically rich in FODMAPs, particularly fructooligosaccharides (FOS), evidence indicates that pasta processing can substantially decrease their content. In fact, cooking and drying conditions may lower the level of FOS by up to 84% [35]. Therefore, careful optimization of technological parameters, such as low-temperature drying, the use of small pasta shapes, and cooking in abundant water, can significantly reduce the FOS content and overall FODMAP burden of rye-based pasta [35].

Alternative grains, such as *Triticum monococcum* (emmer wheat) and *Jermano wheat* (Calabrian rye), have shown potential benefits for IBS management [36]. Furthermore, study indicates that bread made from low-FODMAP rye flour, such as that derived from Jermano wheat, results in decreased colonic fermentation and subsequently reduced flatulence, abdominal pain, and cramping, thus improving patients' quality of life [34].

Our research aims to develop and assess the effects of a functional "green" pasta through preclinical and clinical studies, incorporating ancient grains, specifically emmer wheat (*Triticum monococcum*) and Jermano rye (*Jermano wheat*). The objectives include assessing the impact of this pasta on the symptoms of IBS patients and exploring its potential anti-inflammatory and antioxidant benefits using a cellular model of IBS.

## Materials and methods

### Human study

#### Production of functional pasta

Functional pasta (FP) made from ancient Calabrian grain flours was developed by Astorino Pasta Srl (Crotone, Italy), using a patented formulation (Patent No. 782024000150439, filed 12 September 2024) composed of 80% Jermano rye flour, 10% emmer wheat (Triticum monococcum), and 10% semolina. The production process followed environmentally sustainable ("green") practices, replacing conventional hot water with room-temperature water and reducing mixing time from 30 to 20 min per 50 kg batch. Extrusion was carried out at 60 °C using a commercial press (Pama Roma), and drying occurred at 42 °C for 16–18 h (La Parmigiana Srl), instead of the standard 46 °C. These innovations not only lowered energy use and environmental impact but also improved product performance, resulting in a significantly shorter cooking time of 4–6 min compared to the typical 10–14 min required for traditional pasta. The nutritional composition of the functional pasta is summarized in Table 1.

**Table 1:** Nutritional composition of functional pasta and gluten-free pasta

Nutrient (per 100 g dry weight)	Functional pasta	Gluten-free pasta
Energy (kcal)	311	359
Carbohydrates (g)	62,8	78,7
Protein (g)	9,8	6,5
Total fat (g)	1,5	1,8
Dietary fibre (g)	10,5	1,1

Values are expressed as absolute values per 100 g dry weight, calculated using MetaDieta® software

#### Study design

This is a randomized, open-label clinical trial with a four-week dietary intervention period. The study was conducted at the Clinical Nutrition Unit of the 'Renato Dulbecco' University Hospital in Catanzaro, in collaboration with the Gastroenterology Unit. Patients underwent a comprehensive diagnostic evaluation to exclude organic conditions with overlapping gastrointestinal symptoms. Exclusion criteria included inflammatory bowel diseases (IBD), celiac disease, gastrointestinal infections, neoplasms, food intolerances, and malabsorption syndromes. Once organic causes were ruled out, a gastroenterologist diagnosed IBS according to the Rome IV criteria [15], which require recurrent abdominal pain for at least six months, active in the past three, and associated with at least two of the following: association with defecation, altered stool frequency, or changes in stool form [15]. After enrolment, participants were randomly assigned to one of two dietary intervention groups. The subjects underwent an initial evaluation, during which they were provided with a dietary plan specifying the type of pasta to be consumed (functional pasta or control pasta). The nutritional composition of the functional and control pasta is summarized in Table 1. A final evaluation was conducted after 4 weeks. The study protocol was approved by the local Ethics Committee (Protocol No. 279/2022/CE) and registered in the ISRCTN registry under the identifier ISRCTN12170245. All procedures were conducted in accordance with the principles of the Declaration of Helsinki, and written informed consent was obtained from all participants prior to enrollment. Consecutive subjects with a confirmed diagnosis of IBS, from both genders and aged ≥ 18 years, were enrolled between May 2023 and September 2024. Exclusion criteria encompassed a confirmed diagnosis of celiac disease or wheat allergy, organic gastrointestinal disorders, severe systemic conditions, malnutrition, and psychiatric disorders. Additionally, individuals who had previously adhered to specific dietary regimens were excluded. Pregnant or breastfeeding women were not eligible for participation. At both the initial and final visits, anthropometric parameters were measured, blood samples were collected and analyzed, and adherence to the nutritional plan was assessed. Study participants also completed questionnaires assessing gastrointestinal symptoms and satisfaction with the nutritional treatment.

#### Nutritional intervention duration and assessment

Subjects enrolled in the study were randomized into two treatment groups:

- Group 1: Patients adhering to a low-FODMAP diet that included the FP (80 g, 4 times per week).
- Group 2: Patients adhering to a low-FODMAP diet that included gluten-free pasta (80 g, 4 times per week).

Patients were evaluated at baseline (T0) and again 4 weeks after the start of treatment (T1). Each patient participated in educational sessions and counselling provided by a dietitian to support adherence to a low-FODMAP diet plan. During the initial visit, each patient was provided with a detailed table outlining the foods to be consumed and avoided within each food category (cereals, milk and dairy products, dried fruit, vegetables, legumes, fruit, sweeteners) over the 4-week period. For example, allowed cereals included gluten-free options such as rice, oats, quinoa, and buckwheat, while among legumes, only peas were permitted (Supplemental Table 1). Furthermore, caloric restriction was not recommended for any of the treatment groups, as weight loss was not an objective of this study. Each group received a supply of pasta corresponding to their assigned type (gluten-free or NP), adequate for the 4-week treatment period.

#### Anthropometric measurements

Subjects underwent anthropometric assessments including body weight, measured using a calibrated digital scale (Tanita BC-418MA, Tanita Corp., Tokyo, Japan) accurate to 0.1 kg, and standing height, measured with a stadiometer (Seca 213, Hamburg, Germany) accurate to 0.1 cm. Waist and hip circumferences were also measured to calculate body mass index (BMI). Obesity was defined as a BMI ≥ 30 kg/m^2^.

#### Biochemical assessments

All patients underwent blood sampling. Venous blood was collected using Vacutainer tubes (Becton & Dickinson, Plymouth, England) and centrifuged within 4 h of collection. Serum levels of glucose, triglycerides (TG), total cholesterol (TC), high-density lipoprotein cholesterol (HDL-C), alanine aminotransferase (ALT), aspartate aminotransferase (AST), C-reactive protein (CRP), and creatinine were measured using chemiluminescent immunoassay methods following the manufacturer's instructions on a COBAS 8000 analyzer (Roche, Switzerland). CRP levels were measured at both baseline and follow-up.

#### Clinical questionnaires

At each visit, patients completed a series of validated clinical questionnaires to comprehensively assess their gastrointestinal symptoms, anxiety-depressive symptoms and overall quality of life.

- IBS symptom severity and stool characteristics

To evaluate gastrointestinal symptoms, we used several tools. The IBS—Symptom Severity Score (IBS-SSS) [37] was employed to measure the severity of abdominal pain, distension, satisfaction with bowel habits, and the degree to which these symptoms interfere with daily life. This questionnaire generates a score ranging from 0 to 500, where higher scores denote more severe symptoms. Additionally, the Clinical Evaluation Questionnaire of Bowel Habits [38] was used to assess specific bowel habits, including painful defecation, straining, incomplete evacuation, and stool consistency. Scores for this questionnaire range from 0 to 4, based on the frequency of symptoms. To classify stool types, we utilized the Bristol Stool Form Chart (BSFC) [39], which categorizes stools into types indicating constipation, normalcy, or diarrhea based on visual descriptions.

- Quality of life

The impact of IBS on patients' quality of life was measured using the SF-36 scale (IQOLA SF-36 Italian version 1.6) [40]. This comprehensive questionnaire assesses health status across both physical and mental dimensions, with scores ranging from 0 to 100, where lower scores indicate poorer health and well-being. A cut-off value of 50 is used, with scores above this threshold reflecting a good overall health status, while scores below it indicates impaired general health.

- Anxiety and depression

Anxiety and depressive symptoms were screened using the Hospital Anxiety and Depression Scale (HADS) [41]. This tool provides scores ranging from 0 to 21 for each category, allowing us to categorize the severity of anxiety and depression symptoms.

- Treatment evaluation

Finally, we evaluated the effectiveness of the treatment and the patients' adherence to the prescribed diet. The Evaluation of Improvement and Degree of Satisfaction with Treatment questionnaire rated perceived symptom improvement on a scale from 7 (feels worse) to 1 (responsive to treatment) and measured satisfaction with the low-FODMAP diet on a scale from 0 (not satisfied at all with the diet) to 10 (completely satisfied with the diet) [42]. Additionally, the FODMAP Adherence Report Scale (FARS) [43] assessed diet adherence, with scores ranging from 1 to 5 for each question, and a maximum score of 25 indicating full adherence. These last three questionnaires were administered only during the follow-up visit after the initial assessment.

### In vitro study

#### Extraction and characterization on functional and standard pasta

The FP was ground using a steel blade homogenizer. The powder obtained (36 gr—~ 600 Mesh size) was introduced into a 100 mL tempered steel vial and subjected to extraction with supercritical CO_2_ (SFE-Process—Tomblaine, France). Approximately, 1 kg of CO_2_ was used at ~ 500 bar for the complete extraction procedure and. 20% (w/v) ethanol was used as collecting co-solvent. The same procedure was used for the standard pasta.

#### *ß*-Glucans extraction and quantification of carbohydrate residues

ß-glucans contained in pastas were extracted and characterized as previously described in literature [44]. Phenol–sulfuric acid assay was performed for all carbohydrates determination [45]. Briefly, samples were incubated with sulphuric acid and phenol solution (5% w/v). The obtained mixture was incubated 20 min in ice and absorbance was quantified at λ = 490 nm. Sucrose standard was used as comparison for calibration curve.

#### Proteins content

Proteins content was evaluated by the Bradford assay. In detail, Bradford working reagent was mixed 20 µL samples and absorbance at λ = 595 nm was detected within 30 min of performing the assay. The BSA was used as reference molecule with concentration between 2 and 16 ppm.

#### Total phenolic content (TPC)

Total phenolic content was determined using the Folin-Ciocalteu method [46]. Samples were mixed with the reagent, incubated briefly, then treated with sodium carbonate and incubated again for 25 min in the dark. Absorbance was measured at 760 nm. Gallic acid served as the standard for the calibration curve (0.1–0.5 mg/mL), and results were expressed as milligrams of gallic acid equivalents per milliliter of extract (mg GAE/mL).

#### Total flavonoids content (TFC)

The total flavonoid content in the extract was quantified by a UV–Vis spectrophotometer [47]. Briefly, standard curves were prepared using naringin, apigenin, hesperidin, and rutin dissolved in a DMSO-ethanol mixture. Diluted samples were reacted with sodium nitrite, aluminum chloride, and sodium hydroxide to a final volume of 2 mL. Absorbance was recorded across 200–600 nm to quantify flavonoid levels.

#### Antioxidant activity

Antioxidant activity was assessed using a DPPH-based colorimetric assay [48]. Extracts were incubated with DPPH solution in the dark for 30 min, and absorbance was measured at 518 nm using a UV/VIS spectrophotometer. L-ascorbic acid (5 mg/mL) served as the positive control, and DPPH solution alone as the negative control. Radical scavenging activity was expressed as % inhibition, calculated with the formula: I(%) = [(A0 − A1)/A0] × 100, where A0 is control absorbance and A1 is sample absorbance.

#### HPLC analyses

HPLC analysis was performed using a ThermoFisher Vanquish System with a quaternary pump and UV/VIS detector, managed via Chromeleon® software. Separation was achieved on an Acclaim® 120 C18 reverse-phase column (100 mm × 4.6 mm, 5 µm). The mobile phase consisted of methanol/acetonitrile (2:98), pumped at 0.3 mL/min. Absorbance was recorded at 210–270 nm. Phytosterols were identified by retention time comparison with standards (β-sitosterol, α-tocopherol, stigmasterol, campesterol, brassicasterol). Calibration curves (0.039–1500 ppm) showed r^2^ > 0.99. Each run lasted 65 min.

#### Preparation and characterization of liposomes for in vitro study

To enhance the solubility and stability of FPE bioactive compounds, liposomal encapsulation was used, ensuring better delivery to cells [49]. Liposomes were prepared by thin-layer evaporation followed by extrusion [50]. Briefly, phospholipids (1:1 molar ratio) were dissolved in chloroform, and the solvent was removed via rotary evaporation. FPE obtained with supercritical CO_2_ extraction, was added to the lipid film, then hydrated with MilliQ® water. Large unilamellar vesicles (LUVs) were formed by extrusion at ~ 60 °C using a MiniExtruder®. Vesicle size, distribution, and Z-potential were assessed by dynamic light scattering (Zetasizer NanoZS) using diluted samples in quartz cuvettes [51].

#### Growth and differentiation of Caco-2 and T84 cells

Human colon adenocarcinoma cell line (Caco-2) and colorectal cancer (T84): CCL-284 obtained from ATCC were utilized. Caco-2 cells were cultured in MEM medium supplemented with 20% fetal bovine serum (FBS) while T84 cells were cultured in DMEM-F12 supplemented with 10% FBS. Cells were maintained in a humidified incubator at 37 °C with 5% CO_2_ atmosphere, and the culture medium was changed approximately twice a week to maintain the cultures.

Caco-2 and T84 cells were differentiated into small intestinal enterocytes and colonocytes, respectively [52]. To determine the appropriate stage of differentiation, cells were seeded in 6-well plates at a density of 200,000 cells/well. The culture medium was changed every 2 days.

Gene expression levels of *SI* (*Sucrase-Isomaltase*) and *ALPi* (*Intestinal Alkaline Phosphatase*) in Caco-2 cells, as well as *MS4A12* (*Membrane-Spanning 4-Domains Subfamily A Member 12*) and *SLC16A1* (*Solute Carrier Family 16 Member 1*) in T84 cells, were evaluated at 0, 6, 12, and 18 days of differentiation.

#### Induction of the IBS model and treatment with FPE

To induce in vitro model of IBS [53], Caco-2 and T84 cells were cultured in 6-well plates at a density of 200,000 cells per well once the appropriate differentiation day was confirmed (18 days). The cells were then treated with TNF-α at a concentration of 20 ng/mL and FPE at concentration of 0.25, 0.5, and 1 μg/mL for 24 h. At the end of the incubation, cells and supernatants were collected for viability assays, gene expression, and protein analysis.

#### Cell viability

The cell viability was assessed using the MTT assay. For this assay, 1 × 10^4^ cells/well were seeded in a 96-well dish and treated with FPE at concentrations of 0.25 μg/mL, 0.50 μg/mL, and 1 μg/mL for 24 h. Following treatment, 0.5 mg/mL of MTT solution was added, and the cells were incubated for another 4 h at 37 °C. The formazan crystals formed were then dissolved in 100 μL of dimethyl sulfoxide (DMSO). The absorbance was measured at 570 nm using a microplate reader (Microplate reader 800TS) to quantify the resulting colour intensity.

#### Protein extraction and western blotting

Caco-2 cells were seeded in 35 mm culture dishes at a density of 500,000 cells/well. Cells were lysed in Mammalian Protein Extraction Reagent (M-PER) (Pierce, Thermo Fisher Scientific). Western blot analysis of proteins from cell lysates and from cell medium was performed according to standard procedures. The following antibodies were used: rabbit anti-p Extracellular Signal-regulated Kinase (ERK)1/2 (9101); rabbit anti-NF-kB (Cell Signalling, United States); mouse anti- β-ACTIN (3700).

#### RNA extraction and real-time PCR

Caco-2 and T84 cells were seeded in 35 mm culture dishes at a density of 500.000 cells/well and allowed to differentiate for 6, 12 or 18 days. Samples were extracted according to the TRIzol isolation reagent protocol (Invitrogen, USA), and the quality and quantity of RNA were evaluated by measuring the absorbance at 260 and 280 nm on a NanoDrop Spectrophotometers (Thermo Scientific). The cDNA was synthesized from 1 µg of total RNA, using High-Capacity cDNA Reverse Transcription Kit (Applied Biosystems, Foster City, CA, USA). mRNA expression of *ALPI*, *SI*, *SLC16A1*, *MS4A12*, *NLRP3*, *IL1β*, *IL18*, *GPX1*, *GPX4*, *SOD1* and β-ACTIN were quantified by real time-PCR using SYBR® Green dye (SYBR® Green PCR Master Mix, Applied Biosystems, Foster City, CA, USA) with gene‐specific primers (Supplemental Table 2). Data were compared between samples according to a comparative threshold cycle (2^− ΔΔct^) method and normalized to β‐ACTIN.

**Table 2:** Baseline demographic and clinical characteristics of the population stratified by intervention

Variables	Control (n = 22)	Intervention (n = 20)	*p* value
Age (years)	43 ± 19	45 ± 14	0.78
Weight (kg)	67 ± 13	71 ± 21	0.49
BMI (kg/m^2^)	26 ± 4	27 ± 7	0.35
WC (cm)	87 ± 12	89 ± 21	0.64
Glucose (mg/dL)	92 ± 11	89 ± 9	0.53
Creatinine (mg/dL)	0.83 ± 0.2	0.76 ± 0.1	0.21
TC (mg/dL)	185 ± 33	205 ± 28	0.07
HDL-C (mg/dL)	55 ± 14	66 ± 11	0.018
TG (mg/dL)	116 ± 72	97 ± 44	0.38
AST (U/L)	24 ± 6	26 ± 12	0.56
ALT (U/L)	23 ± 9	31 ± 27	0.26
CRP (mg/L)	1.9 ± 2	2.0 ± 3	0.86
FS—36 scale	56 ± 19	56 ± 19	0.51
HADS-A	9.0 ± 5	8.3 ± 4.3	0.65
HADS-D	6.2 ± 4	5.4 ± 4	0.74
*Prevalence*			
Gender (Female, %)	68	80	0.49
Physical activity (%)	50	45	0.76
Smoking (%)	10	30	0.12
Overweight/Obesity (%)	50	45	0.76
Antihypertensive (%)	23	30	0.73
Lipid-lowering agents (%)	18	5	0.34
Antiplatelet (%)	5	5	1
Oral hypoglycemic agents (%)	5	5	1
FS -36 scale (≥ 50, %)	59	55	1

*BMI* body mass index; *WC* waist circumference; *TC* total cholesterol; *HDL-C* high-density lipoprotein cholesterol; *TG* triglycerides; *AST* aspartate aminotransferase; *ALT* alanine aminotransferase; *CRP* C-reactive protein, *FS-36 scale* 36-item short form survey, *HADS-A* hospital anxiety and depression scale—anxiety subscale A, *HADS-D* hospital anxiety and depression scale—depression subscale D

#### Statistical analysis

For the human study, data are reported as mean ± SD or percentages (%). The sample size was calculated based on a previous study [34], assuming an expected difference of − 10 points in IBS-SSS scores between groups and a common standard deviation of 9.69, yielding an effect size (Cohen’s d) of approximately 1.03. To achieve 80% power at a two-sided 5% significance level, 15 participants per group were required. To account for potential dropouts, a total of 42 subjects were recruited. Participants were randomly assigned to study groups via a computer-generated randomization sequence.

The independent samples t-test and Chi-square test were used to compare differences in means and prevalences between the two groups: those consuming FP and those consuming the gluten-free control pasta. A generalized linear model (GLM) adjusted the IBS-SSS scores for changes in weight at follow-up. Both intention-to-treat (ITT) and *per-protocol* (PP) analyses were conducted. The missing value from the follow-up visit was replaced with the previously observed value for that subject, i.e., the last observation was carried forward [54]. In the ITT analysis, the combination of observed and imputed data was then analyzed as if there were no missing data. Finally, the PP analysis was carried out exclusively on participants who completed the study. Statistical significance was set at *p* < 0.05 (two-tailed). All statistical comparisons were performed using SPSS 29.0 for Windows (IBM Corporation, New York, NY, USA).

For the in vitro study, data are presented as the mean and standard deviation (SD) from at least three independent experiments and analysed using GraphPad Prism 10.0 software with a two-tailed Student’s t-test and linear trend analysis.

## Results

### Human study

Forty-two participants completed the study. The mean age of the enrolled population was 44 ± 16 years. Seventy-four percent of the participants were female, 48% were overweight or obese, and 52% exhibited severe IBS symptoms. Baseline demographic and clinical characteristics of the patients, stratified into the two treatment groups, are reported in Table 2. As shown in the table, the two groups were comparable for all characteristics analysed, except for serum HDL-cholesterol levels, which were significantly higher in the intervention group (*p* = 0.018).

In Table 3, the dietary intake of participants is shown according to the treatment group. At baseline, the nutritional profile of the two groups was comparable.

**Table 3:** The assessment of baseline dietary intake of the population stratified by intervention group

Variables	Control (n = 22)	Intervention (n = 20)	*p* value
Energy (kcal)	1812 ± 458	1912 ± 638	0.56
Carbohydrates (g/day)	218 ± 82	221 ± 89	0.89
Proteins (g/day)	72 ± 17	78 ± 29	0.39
Lipids (g/day)	75 ± 24	82 ± 32	0.44
Saturated fatty acids (g/day)	21 ± 7	24 ± 11	0.23
Monounsaturated fatty acids (g/day)	41 ± 15	44 ± 17	0.57
Polyunsaturated fatty acids (g/day)	10 ± 4	10 ± 4	0.70
Cholesterol (mg/day)	261 ± 100	251 ± 132	0.77
Fiber (g/day)	16 ± 5	19 ± 8	0.19

Table 4 presents the results of the questionnaires regarding bowel habit characteristics and symptom severity. In particular, the two groups were comparable for all analysed characteristics, except for the frequency of abdominal pain reported per week, which was significantly higher in the intervention group (*p* = 0.044). Additionally, no statistically significant differences were observed between the groups regarding the mean IBS-SSS score (*p* = 0.10) or the prevalence of IBS severity symptom (*p* = 0.31).

**Table 4:** Evaluation of bowel habits and IBS-SSS score at baseline in the population stratified by intervention group

Variables	Control (n = 22)	Intervention (n = 20)	*p* value
Abdominal pain severity (%)	48 ± 35	48 ± 26	0.94
Abdominal bloating severity (%)	57 ± 31	73 ± 32	0.12
Satisfaction with bowel habits (%)	72 ± 32	88 ± 21	0.06
IBS-SSS score	280 ± 95	329 ± 81	0.10
IBS-SSS range—Mild (%)	18	10	
IBS-SSS range—Moderate (%)	36	30	0.31
IBS-SSS range—Severe (%)	46	60	
Straining during defecation	1.8 ± 1.3	1.3 ± 1.3	0.21
Incomplete evacuation	2.4 ± 1.3	2.3 ± 1.6	0.71
Painful defecation	0.9 ± 1.2	0.7 ± 1.0	0.54
Hard stools	1.7 ± 1.5	1.3 ± 1.3	0.32
Loose stools	1.8 ± 1.4	2.2 ± 1.4	0.37
Fragmented defecation	1.4 ± 1.4	2.1 ± 1.4	0.12
Urgency to defecate	1.5 ± 1.5	1.4 ± 1.2	0.73
Fecal/gas incontinence	0.2 ± 0.4	0.8 ± 1.4	0.09
Abdominal pain (days/week)	2.0 ± 1.5	3.0 ± 1.3	0.044
Flatulence (days/week)	2.5 ± 1.5	3.2 ± 1.1	0.08
Laxatives/week	0.1 ± 0.4	0.1 ± 0.4	0.77
Enemas and/or suppositories/week	0.2 ± 0.5	0.1 ± 0.2	0.16

*IBS-SSS* IBS—symptom severity score

After 4 weeks of treatment, four patients (two from each group) discontinued participation for personal reasons. FP was well tolerated, with no adverse events. Adherence to the dietary treatment was over 80% in both groups (FARS > 20).

**Fig. 1:**
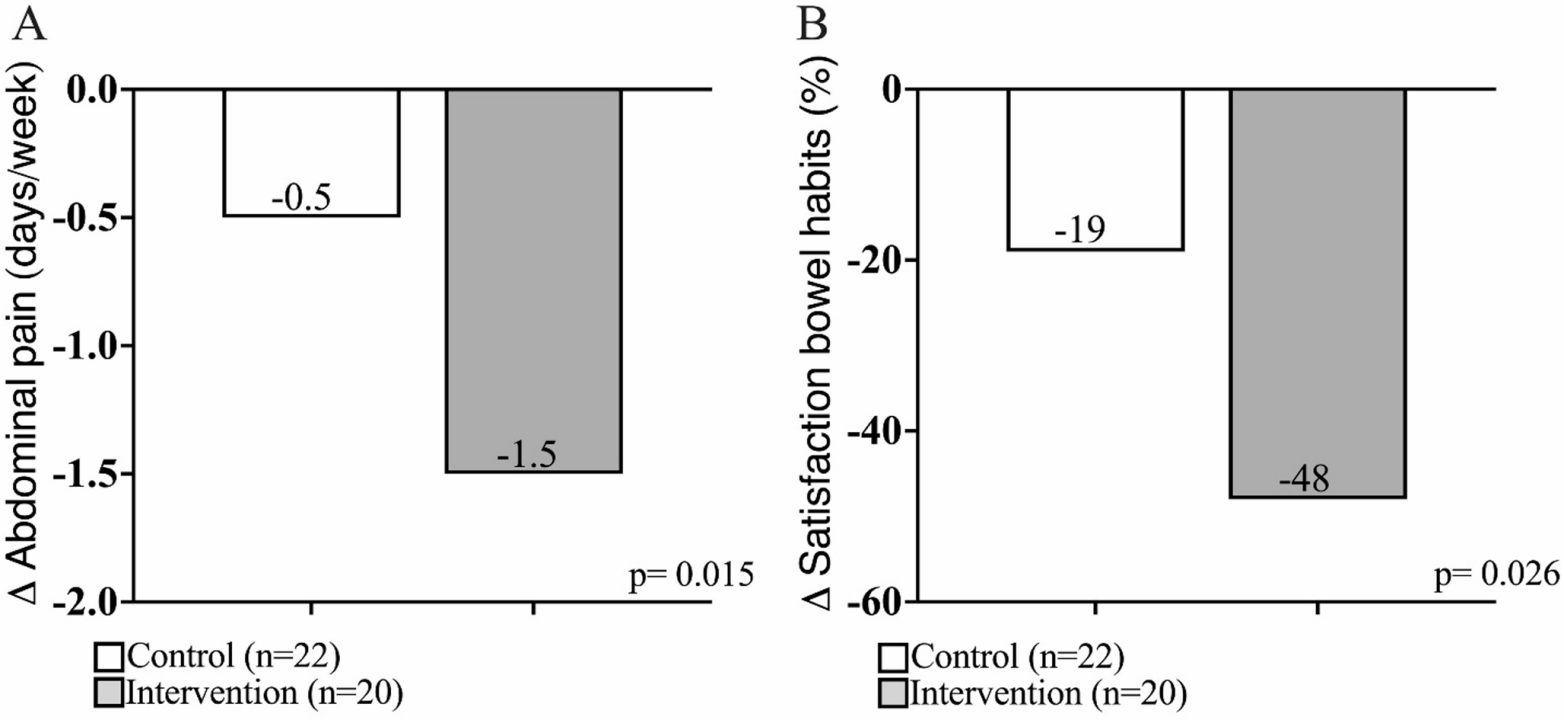
Changes in the weekly frequency of abdominal pain (**A**) and satisfaction with bowel habits (**B**) after 4 weeks of FP intervention

The group consuming the FP showed a statistically significant reduction in the weekly frequency of abdominal pain (*p* = 0.015) and greater satisfaction with bowel habits (*p* = 0.026) compared to the control group (Fig. 1).

No other statistically significant differences were observed between the groups regarding anthropometric parameters, bowel habits, bowel management treatments, or other evaluated parameters, including physical functioning, anxiety, and depressive symptoms.

Further details, including a full evaluation of the results, are presented in Supplemental Table 3.

**Fig. 2:**
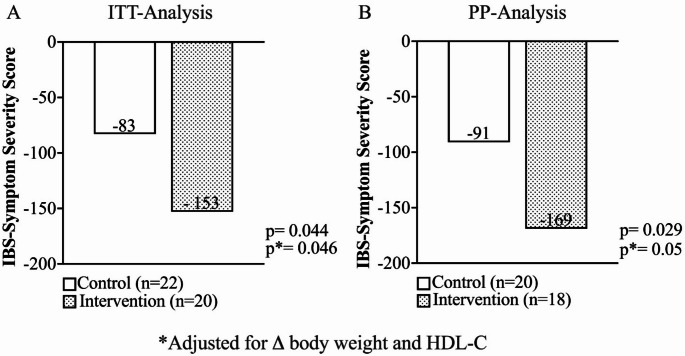
Reduction in the IBS-SSS score after 4 weeks in the population stratified by treatment group. **A** ITT (intention-to-treat) analysis and **B** PP (per-protocol) analysis

Figure 2 shows the change in the IBS-SSS score between the two treatment groups after 4 weeks, both in the ITT and PP analyses. As shown in the figure, patients who consumed FP demonstrated a statistically significant reduction in the IBS-SSS score compared to the control group [− 153 ± 124 (CI − 210; − 94) (− 46%) vs. − 83 ± 85 (CI − 121; − 45) (− 28%), *p* = 0.044, respectively]. This reduction remains statistically significant even after adjusting for changes in body weight at follow-up and HDL-C at baseline [− 163,1 ± 27 (CI − 139; − 36) vs − 87 ± 25 (CI − 223; − 113), *p* = 0.046, respectively]. Moreover, a greater statistically significant reduction in the IBS-SSS score was also confirmed in the PP analysis for the group consuming FP, compared to the control group [− 169 ± 119 (− 51%) (CI − 229; − 110) vs. − 91 ± 85 (CI − 131; − 52) (− 31%), *p* = 0.029, respectively]. Again, this reduction remains statistically significant after adjusting for changes in body weight at follow-up and HDL-C at baseline [− 180 ± 27 (CI − 237; − 123) vs. − 99 ± 26 (CI − 153; − 44), *p* = 0.05, respectively].

**Fig. 3:**
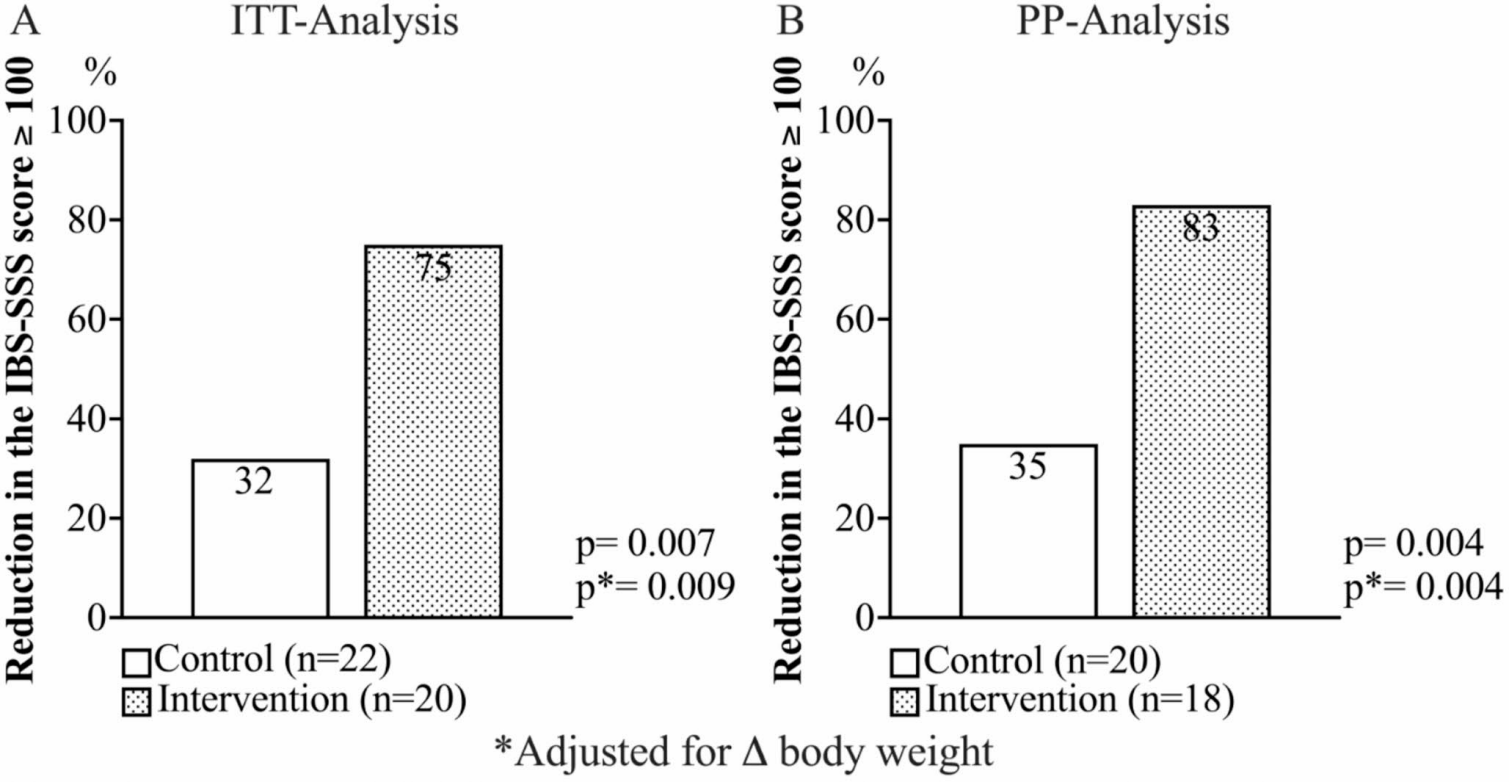
Reduction in IBS-SSS score (≥ 100 points), after 4 weeks, in the control and intervention groups. **A** ITT (intention-to-treat) analysis and **B** PP (per-protocol) analysis

Figure 3 shows the responder rate, defined as the percentage of subjects who, after 4 weeks of treatment, reported a reduction in the IBS-SSS score ≥ 100. Both in the ITT and PP analyses, a higher percentage of responders was observed in the group that consumed FP compared to the control group [ITT: 75% vs. 32%, *p* = 0.007; PP: 83% vs. 35%, *p* = 0.004, respectively]. These results remain significant even after adjusting for changes in body weight at follow-up (Fig. 3).

**Fig. 4:**
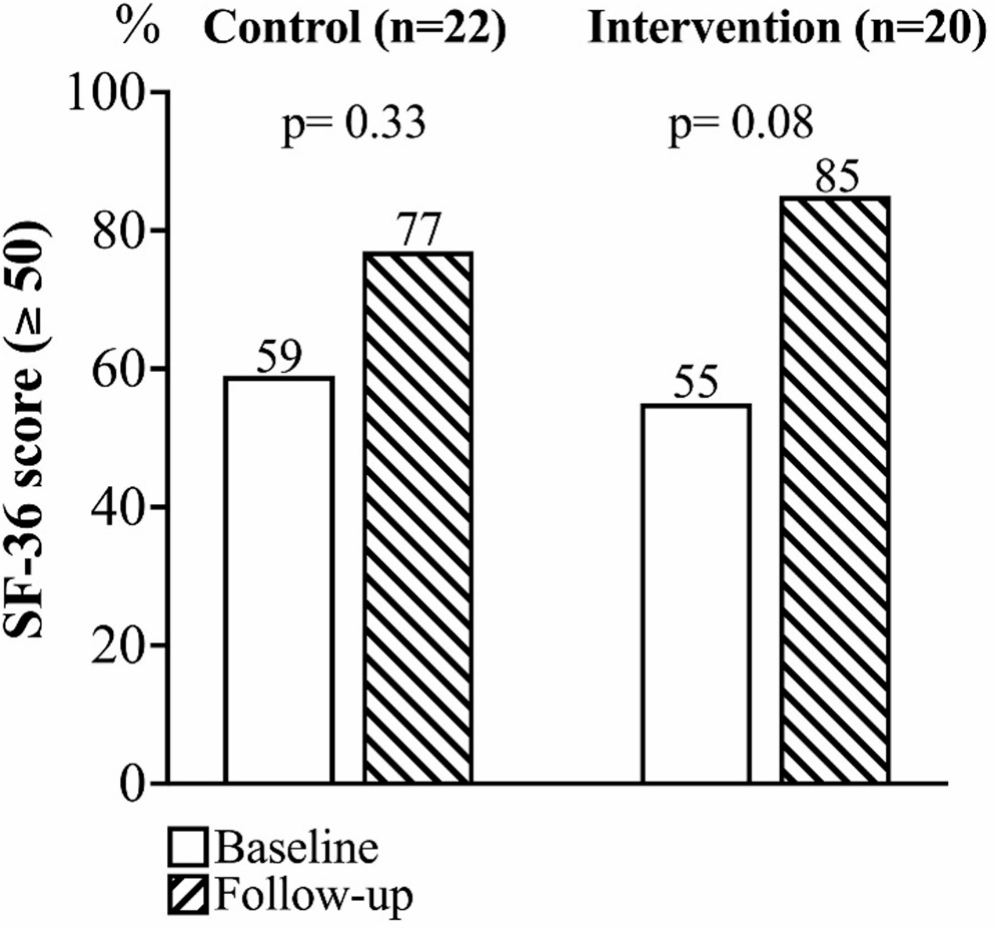
Change in prevalence of subjects with an SF-36 score > 50 in the control and intervention groups from baseline to the 4-week follow-up

Finally, at follow-up, the prevalence of subjects with a good overall health status (SF-36 score ≥ 50) was 77% in the control group and 85% in the intervention group (*p* = 0.70). Figure 4 illustrates the change in prevalence within each group, showing a non-statistically significant trend toward an increased proportion of subjects with an SF-36 score > 50 in the group consuming the FP at follow-up compared to baseline (85% vs. 55%, *p* = 0.08).

### In vitro study

#### Comparison FPE with standard pasta extract and liposomal suspension

**Fig. 5:**
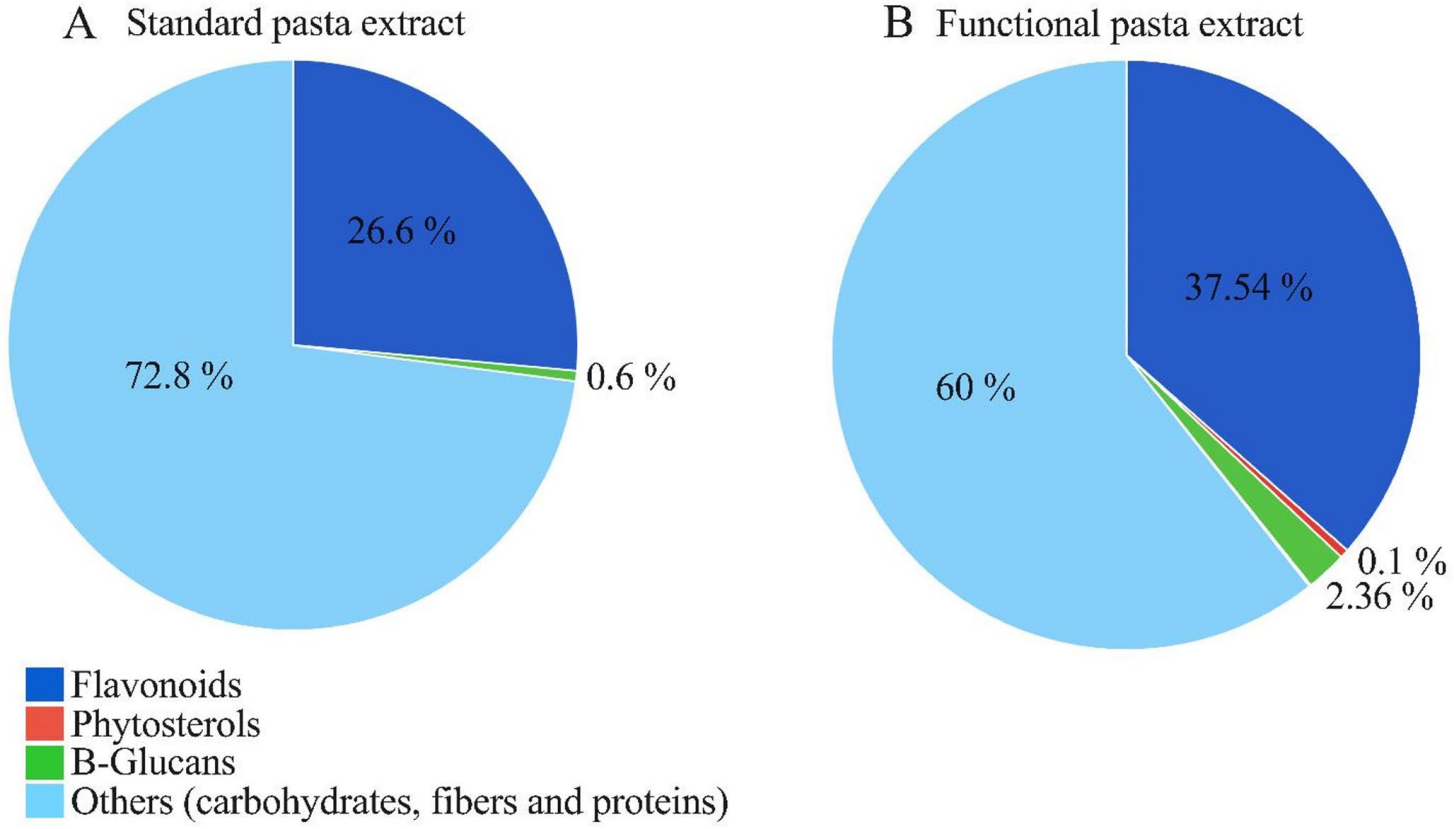
Comparative composition of Standard and Functional Pasta Extracts. **A** Composition of **A** standard and **B** functional pasta extract

Figure 5 demonstrates that, per 100 g, the functional pasta extract (FPE) contains flavonoids (FPE: 37.54% *vs*. SPE: 26.6%), which provide an antioxidant activity of 47.16 ± 0.14% inhibition, expressed as the capacity to inhibit free radicals, and β-glucans (FPE: 2.36% in *vs*. SPE: 0.6%). Furthermore, the FPE has a total carbohydrate, fiber, and protein content of 60%, compared to 72.8% in SPE. Finally, the FPE contains 0.1% phytosterols, which are absent in SPE.

To enhance phytosterol delivery, a liposomal formulation was developed. FPE incorporation increased vesicle size (from 159.3 nm to 280.2 nm) and reduced surface charge (+ 13.2 mV to + 4.23 mV), confirming successful encapsulation while maintaining acceptable dispersion properties Supplemental Fig. 1 and Supplemental Table 4).

#### Caco-2 and T84 cell differentiation into small intestinal enterocytes and colonocytes after 18 days

**Fig. 6:**
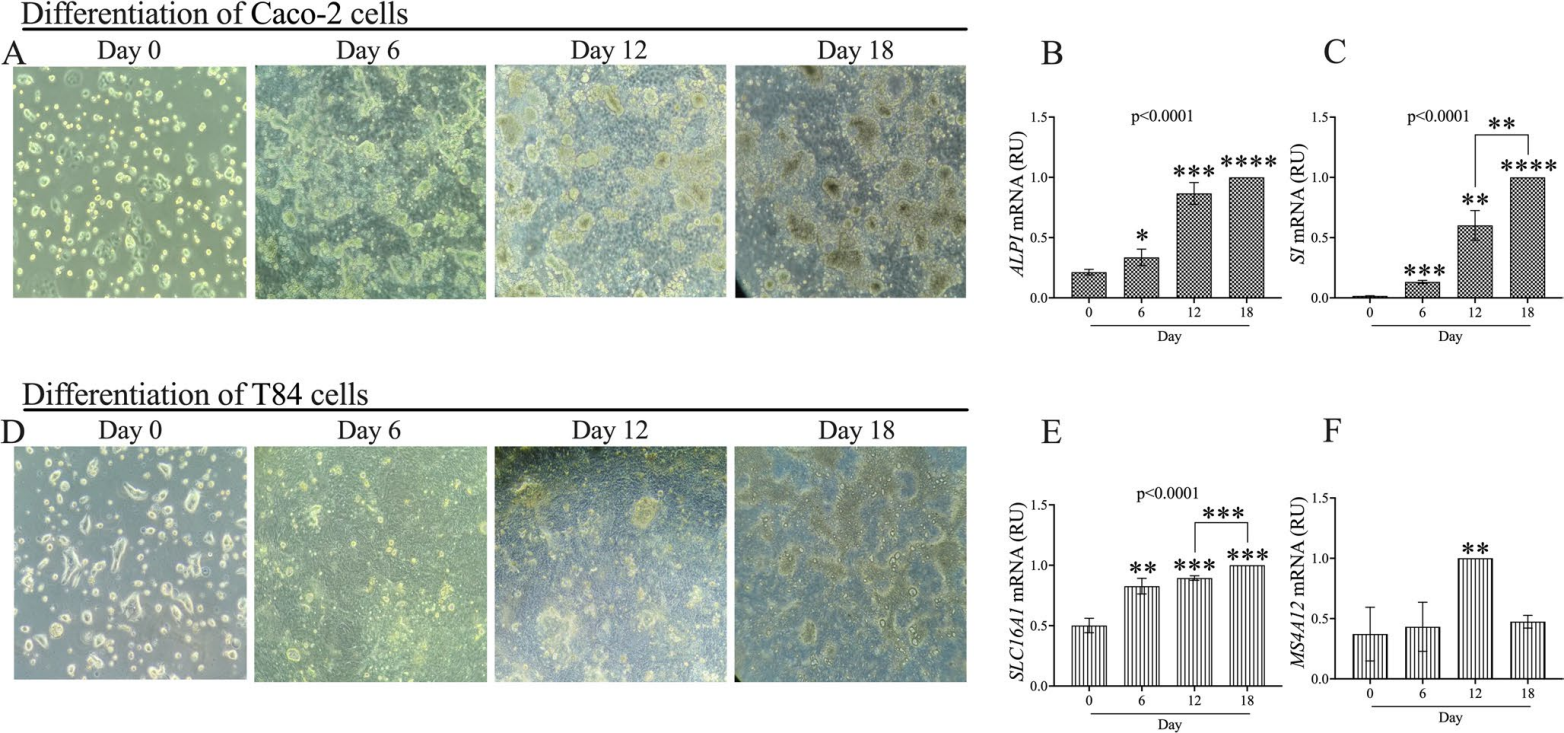
Caco-2 and T84 differentiation to small intestinal enterocytes and colonocytes over 18 days. **A**, **D** Morphological changes in Caco-2 and T84 cells observed at Day 0, 6, 12, and 18, demonstrating progressive differentiation into small intestinal enterocytes and colonocytes, respectively. **B**, **C**, **E**, **F** mRNA expressione of ALPI, SI, SLC16A1 and MS4A12 were measured using Real-Time PCR. Data were analyzed using the 2^^−ΔΔCt^ method and normalized to β-ACTIN. Data are represented as mean ± SD of three independent experiments. *p* values calculated by Student’s t-test: *p < 0.05, **p < 0.01; ***p < 0.001; ****p < 0.0001. Abbreviations: RU = Relative unit

To assess the optimal differentiation time of Caco-2 and T84 cells into small intestinal enterocytes and colonocytes, respectively, differentiation was evaluated at multiple time points (0, 6, 12, and 18 days). As shown in Fig. 6B and C, the expression levels of *SI* and *ALPi* in Caco-2 cells significantly increased by day 18 compared to day 0 (*p* < 0.0001 and *p* < 0.0001, respectively). In T84 cells differentiated for 18 days, there was a statistically significant increase in the expression of *SLC16A1*, whereas no modulation was observed in the *MS4A12* gene after 18 days (Fig. 6E, F).

#### Efficacy of FP extract in attenuating TNF-α-induced inflammation and oxidative stress in Caco-2 and T84 cell lines

**Fig. 7:**
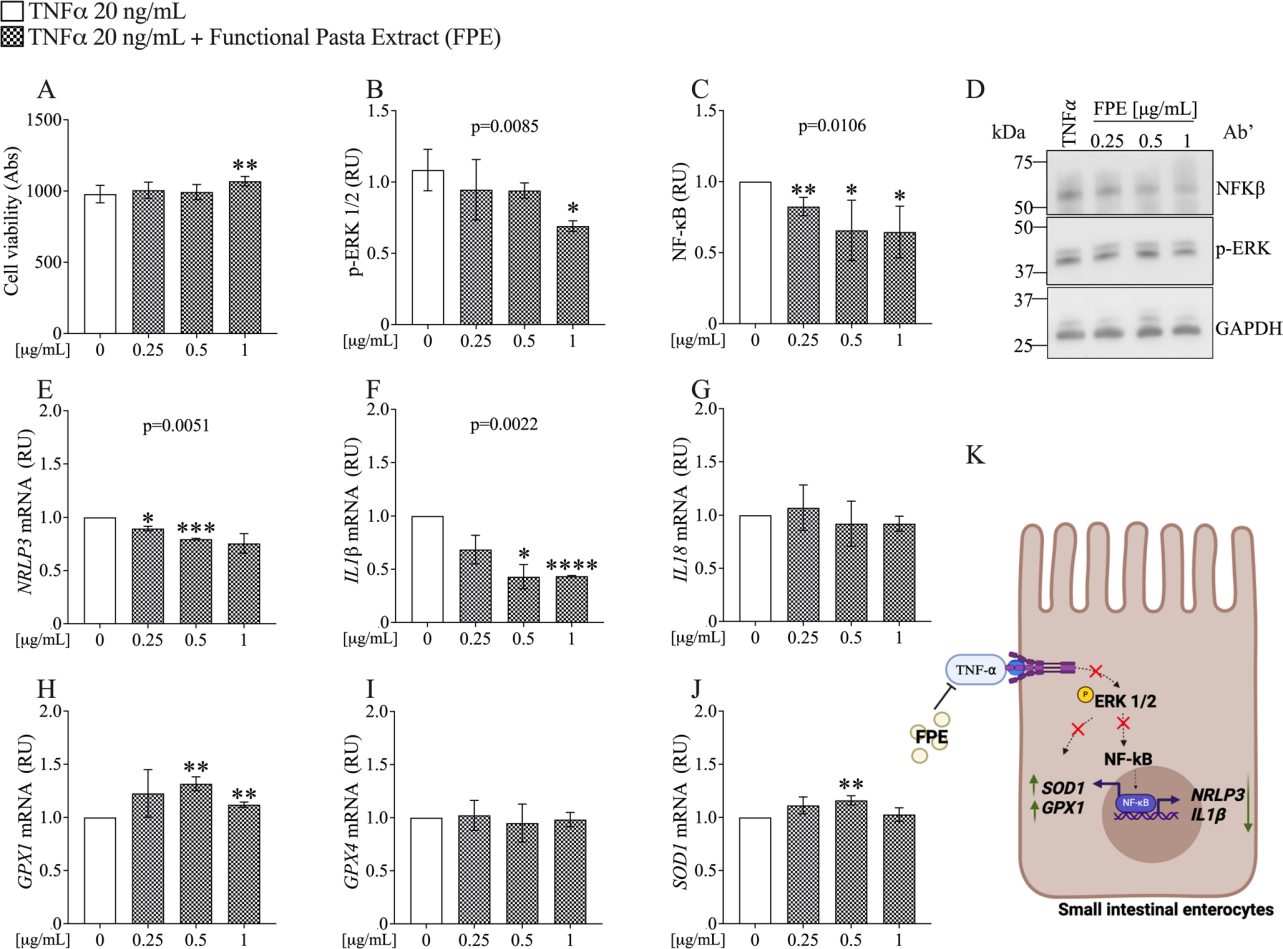
FPE Attenuates TNF-α-Induced Inflammation and Oxidative Stress in Small Intestinal Enterocytes. **A** Assessment of cell viability by MTT assay in Caco-2 cells. **B**–**D** Protein levels by western blotting and relative quantification of p-Erk1/2 and NF-κB expression. **E**–**J** mRNA expression levels of NRLP3, IL-1b, IL18, GPX1, GPX4 and SOD1 were measured using Real-Time PCR. Data were analyzed using the 2^^−ΔΔCt^ method and normalized to β-ACTIN. Data are represented as mean ± SD of three independent experiments and p-values are calculated by Student’s t-test: 0 (TNF-a) vs FPE *p < 0.05, **p < 0.01; ***p < 0.001; ****p < 0.0001. Abbreviations: RU = Relative Unit

To ensure that the doses of the extract were not toxic to Caco-2 cells, we performed a cell viability assay using the MTT method, treating the cells with doses of 0.25, 0.5, and 1 µg/mL of FPE. Results depicted in Fig. 7A show that at 1 µg/mL, FPE significantly increases cell viability, demonstrating the extract's safety.

To test the hypothesis that FPE exerts an inhibitory effect on pathways involved in TNF-α-induced inflammation and oxidative stress, the protein expression of Erk1/2 and NF-κB was evaluated (Fig. 7). Data shown in Fig. 7B, C demonstrate that FPE treatment significantly reduces the expression of p-Erk1/2 and NF-κB in a dose-dependent manner compared to TNF-α (*p* = 0.0085 and *p* = 0.0106, respectively).

Figure 7E and F show that FPE treatment leads to a significant, dose-dependent decrease in the gene expression of the *NLRP3* inflammasome and *interleukin-1β (IL-1β)* compared to controls treated with TNF-α (*p* = 0.0051 and *p* = 0.0022, respectively). No significant effect of FPE was observed on *interleukin-18* (*IL-18*) expression (Fig. 7G).

Figure 7H–J present data on the gene expression of oxidative stress markers. Specifically, FPE was found to enhance the expression of *GPX1* at concentrations of 0.5 and 1 µg/mL (Fig. 7H), while a reduction in *SOD1* expression was noted at only the 0.5 µg/mL dose following FPE treatment compared to TNF-α (Fig. 7J). These findings collectively demonstrate that FPE can attenuate TNF-α-induced inflammation and oxidative stress in Caco-2 cells differentiated into small intestinal enterocytes. Figure 7K illustrates the effects of FPE in small intestinal enterocytes.

**Fig. 8:**
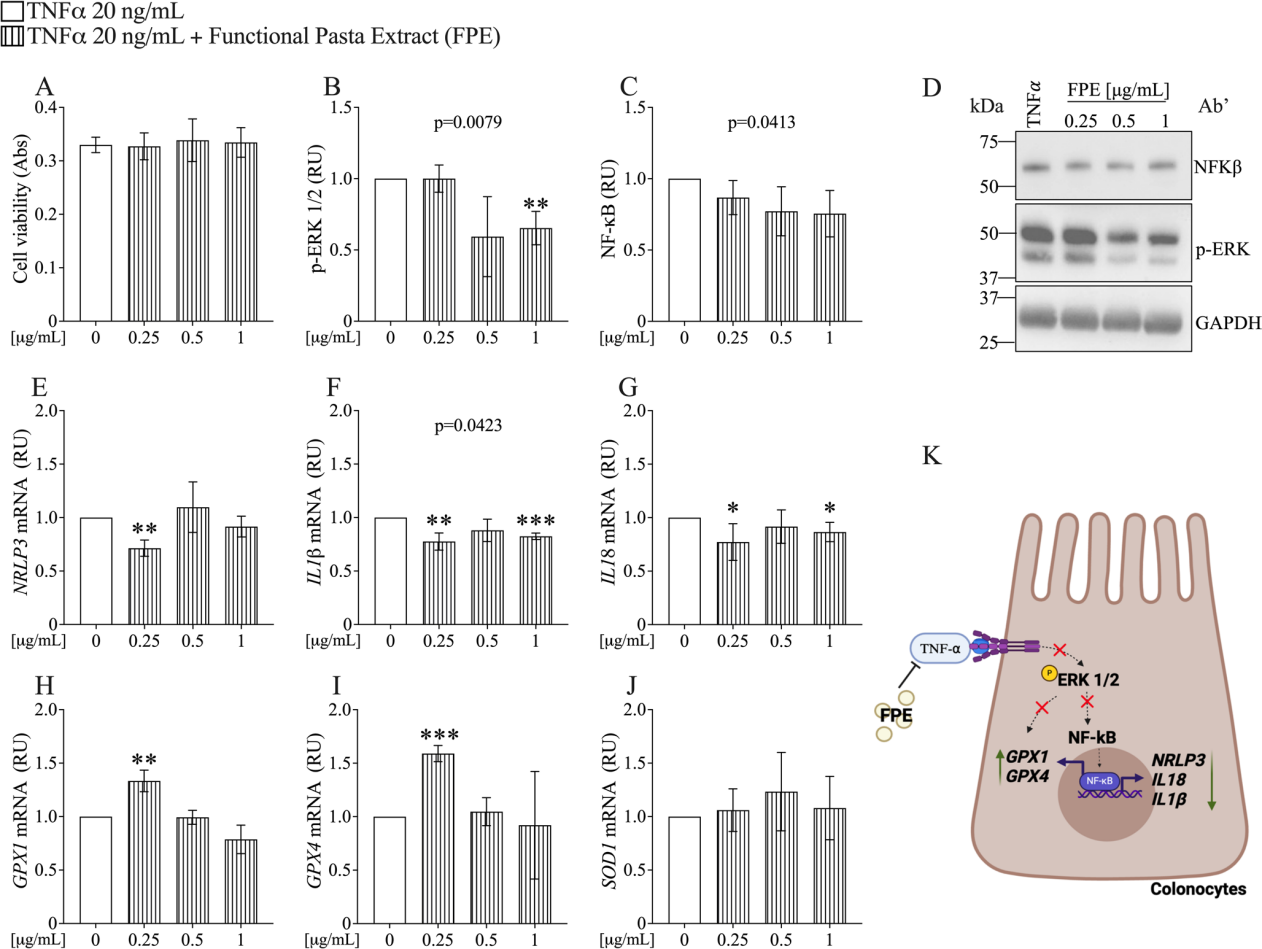
FPE reduce TNF-α-induced inflammation and oxidative stress in colonocytes. **A** Assessment of cell viability by MTT assay in T84 cells. **B**–**D** Protein levels by western blotting and relative quantification of p-Erk1/2 and NF-κB expression. **E**–**J** mRNA expression levels of *NRLP3*, *IL*-*1b*, *IL18*, *GPX1*, *GPX4* and *SOD1* were measured using Real-Time PCR. Data were analyzed using the 2^^−ΔΔCt^ method and normalized to β-*ACTIN*. Data are represented as mean ± SD of three independent experiments and *p*-values are calculated by Student’s t-test: 0 (TNF-a) vs FPE *p < 0.05, **p < 0.01; ***p < 0.001. Abbreviations: RU = Relative Unit

To ensure that the doses of the extract were not toxic also to T84 cells, we performed a cell viability assay using the MTT method, treating the cells with doses of 0.25, 0.5, and 1 µg/mL of FPE. Figure 8A demonstrates that the FPE extract does not adversely affect cell viability.

To assess the anti-inflammatory and antioxidant effects of FPE on colonocytes, we investigated the pathways involved in TNF-α-induced inflammation and oxidative stress (Fig. 8). Figure 8B, C indicate that FPE treatment led to a significant, dose-dependent reduction in the expression of p-Erk1/2 and NF-κB compared to treatment with TNF-α (*p* = 0.0079 and *p* = 0.0416, respectively).

Additionally, Fig. 8E–G shown that FPE treatment led to a significant decrease in the gene expression of the *NLRP3* inflammasome at a dose of 0.25 µg/mL, and IL-1β and IL-18 at doses of 0.25 and 1 µg/mL, compared to controls treated with TNF-α.

Regarding oxidative stress markers, we observed that FPE enhances the expression of *GPX1* and *GPX4* at a concentration of 0.25 µg/mL compared to TNF-a (Fig. 8H, I). Not change was observed in *SOD1* expression (Fig. 8J). These findings demonstrate that FPE can attenuate TNF-α-induced inflammation and partially oxidative stress in colonocytes. Figure 8K highlights the effects of FPE in colonocytes.

## Discussion

In the context of the growing emphasis on sustainable agricultural practices and the search for climate-resilient crops, there has been significant interest in using ancient grain flours as functional ingredients in food production, not only for their well-recognized nutritional and health benefits but also for their lower environmental impact and potential to enhance global food availability [55–58].

The objective of this study was to develop and evaluate a 'green' functional pasta incorporating *Triticum monococcum* (Emmer) and *Jermano wheat* (Rye). Indeed, compared to traditional pasta, the production of FP not only follows environmentally sustainable practices but also entails lower manufacturing costs. The optimized formulation and processing conditions, such as reduced water temperature, shorter mixing times, and lower drying temperatures, contribute to decreased energy consumption and operational expenses. Additionally, the significantly reduced cooking time (4–6 min vs. 10–14 min for conventional pasta) further enhances its cost-effectiveness, making it a viable and sustainable alternative for large-scale production.

Specifically, the study investigated the effects of the pasta on the clinical symptoms of patients with IBS and examined the potential anti-inflammatory and antioxidant properties using an in vitro cellular model. To further elucidate the therapeutic potential of the pasta, a randomized, open-label clinical trial was conducted to assess its impact on gastrointestinal symptoms in 42 IBS patients. Our results suggest that the consumption of this functional pasta can lead to a significant reduction in the IBS-SSS score, indicating an improvement in overall symptoms, including abdominal pain frequency and severity. The baseline characteristics of the two treatment groups were comparable. The two groups also exhibited similar dietary intake profiles at baseline, suggesting that any differences observed in gastrointestinal symptoms can be attributed to the intervention rather than baseline dietary habits. In terms of gastrointestinal health, the intervention group experienced a significant reduction in the frequency of abdominal pain (*p* = 0.015), and improved satisfaction with bowel habits (*p* = 0.026) after 4 weeks of treatment. This is particularly relevant for IBS patients who often report difficulty in managing symptoms such as abdominal pain and bloating [59]. Unlike most other IBS symptoms, such as bloating or changes in stool frequency or form, abdominal pain independently impairs health-related quality of life and is a major contributor to the severity of patient-reported symptoms [60]. In essence, IBS is partly characterized by pain, which plays a central role in the disease experience for many patients [61, 62]. Although no significant differences were observed in other bowel characteristics, the reduction in abdominal pain and improved bowel satisfaction are noteworthy outcomes that may have a meaningful clinical impact.

Interestingly, the intervention group demonstrated a higher responder rate in the IBS-SSS score reduction compared to the control group (75% vs. 32%, *p* = 0.007). This suggests that the FP may have a more pronounced effect in alleviating IBS symptoms in a substantial proportion of patients, which supports its potential as a functional food for managing severe IBS.

In our study, we observed no significant differences between the intervention and control groups in terms of physical functioning, anxiety levels or depressive symptoms. These findings align with previous research indicating that while certain dietary interventions can alleviate specific gastrointestinal symptoms in IBS patients, they may not uniformly impact all aspects of health-related quality of life or psychological well-being. For instance, a randomized controlled trial by Eswaran et al. [24] found that a low FODMAP diet led to significant improvements in individual IBS symptoms, particularly pain and bloating, but did not observe significant changes in anxiety or depression scores. Similarly, Halmos et al. [63] reported that a diet low in FODMAPs effectively reduced functional gastrointestinal symptoms without significantly affecting psychological parameters. These results suggest that while the functional pasta may specifically alleviate key gastrointestinal symptoms such as abdominal pain and enhance bowel satisfaction, its influence on general physical function and psychological parameters appears limited within the four-week intervention period. It is possible that these broader aspects of health may require longer-term dietary modifications to manifest measurable changes.

Our findings are in line with previous research showing that dietary interventions, such as the inclusion of functional foods rich in ancient grain flours, can improve gastrointestinal health in IBS patients [13, 34]. A double-blind, randomized crossover study investigated the effects of a diet based on semi-whole organic products made from the ancient wheat *Triticum turgidum turanicum* on IBS symptoms [13]. The ancient wheat intervention significantly improved IBS symptom severity, including abdominal pain, bloating, stool consistency satisfaction, and fatigue, with no significant changes observed after the “modern” wheat period [13]. However, our study provides additional insights beyond previous research. First, our functional pasta was produced using an eco-friendly manufacturing process with lower energy consumption, reduced processing time, and lower-temperature drying, contributing to a more sustainable food system. Second, while previous studies have primarily investigated other ancient wheat varieties, such as Triticum turgidum turanicum, our research examines a unique combination of the less-explored *Triticum monococcum* and *Jermano wheat*, both deeply embedded in the agricultural heritage of southern Italy. This aspect enhances the value of our findings in terms of biodiversity conservation and the promotion of regional food heritage. Notably, *Triticum monococcum and Jermano wheat,* the ancient grains examined in our study, has been reported to contain gluten with lower immunogenic potential and greater digestibility compared to modern wheat varieties [64, 65]. Moreover, the ancient grains we investigated exhibit a distinct gluten quality, characterized by a weaker and less elastic structure [64]. A study by De Santis et al. [66] compared traditional wheat cultivars with modern ones, focusing on glutenin and gliadin content, including the immunogenic α- and γ-gliadins. While levels of α- and γ-gliadins remained stable in modern wheat, ω-5 gliadin levels decreased. However, modern cultivars showed higher glutenin content, reducing the gliadin-to-glutenin ratio from 2.8 to 1.7. This suggests that selective breeding for stronger gluten may have inadvertently reduced digestibility. Notably, ‘toxic’ gluten peptides are generated during gastric and intestinal digestion. If gliadins and glutenins were completely hydrolyzed, their immunogenic potential would be significantly reduced. In fact, the gluten strength of modern wheat, as indicated by gluten strength (W) values, is typically two to three times higher than that of its predecessors. This difference stems from the selective breeding practices implemented since the 1940s–1950s, where high W values became a key criterion in wheat selection [67]. Finally, unlike previous studies that primarily assessed clinical outcomes, we also explored the potential anti-inflammatory and antioxidant properties of this pasta in a cellular model of IBS, providing a mechanistic explanation for its beneficial effects.

Overall, while the ancient wheat pasta did not lead to significant changes in body weight or other anthropometric parameters, the positive effects on gastrointestinal health, particularly the reduction in IBS-SSS scores, highlight its potential as a therapeutic dietary intervention for IBS management.

To better understand how our functional pasta exerts beneficial effects in individuals with IBS, we conducted in vitro tests on the functional pasta extract. The data from our study indicate that the FPE contains higher concentrations of flavonoids (+ 37.5%) and β-glucans (+ 2.3%) compared to standard pasta, as well as lower quantities of carbohydrates and proteins. Additionally, we have detected and quantified the exclusive presence of phytosterols (0.1% w/w) and polyphenols linked to gallic acid (< 0.1% w/w) in the ancient grain pasta. These compounds are known for their antioxidant and anti-inflammatory effects, contributing to improved cardiovascular health and metabolic function [68, 69]. Moreover, β-glucans have been shown to support gut health by promoting beneficial microbiota and enhancing the intestinal barrier, which may help alleviate symptoms of IBS [70, 71]. This highlights the potential of ancient grain pasta as a functional food that not only provides nutritional benefits but also promotes digestive health, particularly in individuals with IBS. Notably, its high β-glucan content may contribute to symptom relief, as Asano et al. demonstrated that β-glucans can attenuate visceral pain responses to colorectal distension in vivo models of IBS [72].

For the first time, we demonstrate that FPE protects intestinal enterocytes (Caco-2) and colonocytes (T84) from inflammation and oxidative stress induced by TNF-α, a key cytokine in the pathogenesis of inflammatory bowel diseases. Previous studies have shown that TNF-α activates signaling pathways such as NF-κB and ERK1/2, which not only promote inflammatory processes but also compromise the integrity of the intestinal barrier [62, 73]. Our results reveal that FPE can negatively modulate these pathways, confirming its anti-inflammatory potential and further demonstrating its ability to inhibit the gene expression of the *NLRP3* inflammasome and the inflammatory cytokines *IL-1β* and *IL-18*, thereby underlining its effectiveness. Additionally, we demonstrate that our extract is capable of modulating enzymes involved in cellular protection from oxidation and oxidative stress, such as *GPX1*, *GPX4*, and *SOD1*, particularly in Caco-2 cells. These mechanistic effects provide a strong rationale for the clinical findings: the observed reduction in abdominal pain and the improvement in bowel satisfaction are consistent with the capacity of polyphenols and flavonoids to attenuate ROS generation and oxidative stress–induced signaling, inhibit NF-κB/ERK1/2 activation, and preserve tight junction proteins such as occludin, claudin-1, and ZO-1 [74, 75]. In parallel, β-glucans can interact with Dectin-1 and TLR-2/6 receptors on intestinal epithelial and immune cells, promoting anti-inflammatory cytokine release, strengthening mucosal barrier integrity, and potentially reducing visceral hypersensitivity [76, 77]. Together, these mechanisms explain how FPE bioactives synergistically protect intestinal homeostasis, thereby translating into measurable symptom relief in IBS patients.

This is further supported by the unique composition of FPE, particularly its enrichment in polyphenols, flavonoids, and β-glucans, whose combined actions may underlie the observed effects. Literature indicates that flavonoids, particularly *flavone-O-glycosides* found in rye [78], possess significant anti-inflammatory and antioxidant properties [78, 79]. Moreover, flavonoids are known to play a role in regulating the integrity of the tight junction (TJ) barrier in human intestinal Caco-2 cells [80]. In particular, it has been demonstrated that flavonoids can prevent TNFα-induced permeabilization by inhibiting ERK1/2 and NF-κB pathways, which are dependent on NADPH oxidase activity [81]. These effects are more pronounced in enterocytes compared to colonocytes, likely because Caco-2 cells, which model enterocytes, exhibit greater nutrient absorption capacities, thereby enhancing the uptake of polyphenols/flavonoids [52, 82]. Instead, β-glucans have been shown to reduce colitis both at molecular and organ levels, accelerating the remission of chronic intestinal diseases [83]. Moreover, a placebo-controlled trial utilizing a mushroom extract rich in β-glucans reported modest improvements in quality of life and inflammatory markers in IBS patients [84]. Further studies confirm that β-glucans can protect against intestinal inflammation and reduce pro-inflammatory markers, suggesting a significant therapeutic potential [85, 86]. These findings support the hypothesis that bioactive compounds such as polyphenols and β-glucans significantly contribute to the efficacy of functional pasta in modulating inflammatory responses and oxidative stress, both in vitro and in patients with IBS.

These findings reinforce the role of this functional pasta as a promising dietary strategy for IBS management while supporting the integration of sustainable food innovations into clinical practice. In addition, the production of this functional pasta aligns with sustainable and environmentally friendly practices. The manufacturing process minimizes energy consumption by utilizing ambient temperature water, reducing mixing and drying times, and operating at lower extrusion and drying temperatures. Moreover, the significantly shorter cooking time (4–6 min vs. 10–14 min for traditional pasta) further decreases energy use at the consumer level. By integrating these 'green' practices, this pasta represents a step toward more sustainable food production while preserving the nutritional and functional properties of ancient grains. Beyond its potential health benefits, the introduction of functional pasta made from ancient Calabrian grains, naturally richer in dietary fiber than the control pasta, reflects the principles of the Mediterranean diet. By incorporating *Triticum monococcum* and *Jermano*, this pasta offers nutritional advantages and supports adherence to a traditional, balanced dietary pattern, which has been associated with improved gut health and reduced inflammation [87, 88]. However, patients following a low-FODMAP diet may need to limit certain Mediterranean staples, such as legumes and some fruits, when planning dietary interventions for IBS [88].

This study has several limitations. First, we did not perform a statistical analysis of the intervention's efficacy across IBS subgroups due to the small sample sizes within each subtype. The limited number of patients in each category restricted our ability to conduct meaningful subgroup comparisons. However, the intervention was tested across the entire study population regardless of IBS subtype, and its overall efficacy was demonstrated. Future studies with larger, well-stratified cohorts would be beneficial to assess potential variations in response across different IBS subtypes.

The open-label, non-blinded design of the clinical trial introduces the potential for observer bias, which may affect the objectivity of the outcome assessments. The two types of pasta (standard and ancient grain) were visually different. Nevertheless, the in vitro study results confirm the clinical data, and the reduction in symptoms can be attributed to the functional pasta.

Another limitation is that we did not directly quantify the FODMAP content of the functional pasta; however, compositional analysis of its bioactive fraction, literature evidence on ancient rye cultivars, and the additional reduction of FOS during the cooking process [35] all support the plausibility of a lower FODMAP profile, consistent with the clinical outcomes observed.

In addition, baseline HDL-C levels were higher in the intervention group. Importantly, after adjusting for HDL-C, our results remained statistically significant, confirming the robustness of the findings and reducing the likelihood of confounding.

Moreover, we did not assess systemic markers of inflammation, microbiota composition, or broader quality-of-life outcomes. Nevertheless, the parallel in vitro experiments provide mechanistic evidence that reinforce the plausibility of the observed clinical effects, partially compensating for the lack of systemic measurements.

Finally, although the sample size is limited, the statistical analysis remains valid, as demonstrated by other studies with a similar number of participants [13]. The study's applicability is somewhat restricted due to its short duration; however, it serves as a solid foundation for designing longer-term studies that could further validate these findings and provide a more comprehensive understanding of the effects over time.

## Conclusions

In conclusion, our study demonstrates that innovative pasta made from ancient Calabrian grains, *Triticum monococcum* and *Jermano, holds* promise as a functional food with the potential to alleviate symptoms such as abdominal pain and improve bowel satisfaction in IBS. The findings suggest that this "green" pasta, produced using sustainable practices, not only offers nutritional and functional benefits but also aligns with the growing demand for eco-friendly, climate-resilient food options. This research highlights the potential for traditional, locally sourced ingredients to play a pivotal role in modern nutrition and healthcare, supporting both individual health and environmental sustainability.

While the results are encouraging, further research with controlled trial designs, larger sample sizes, and longer follow-up periods is necessary to confirm the long-term effectiveness and sustainability of these benefits.

## Supplementary Information

Below is the link to the electronic supplementary material.

Supplementary Material 1

Supplementary Material 2

Supplementary Material 3

Supplementary Material 4

## Data Availability

Data presented in the manuscript will be made available upon request pending approval.
