# Supplementary material for: Ancient grain flour consumption as a novel therapeutic approach for irritable bowel syndrome
Source: Eur J Nutr. 2025 Dec 19;65(1):11. doi: 10.1007/s00394-025-03859-8 (PMC12717202; doi:10.1007/s00394-025-03859-8)
Supplement: Supplementary file 1 — Supplementary Material 1 [file 394_2025_3859_MOESM1_ESM.docx]

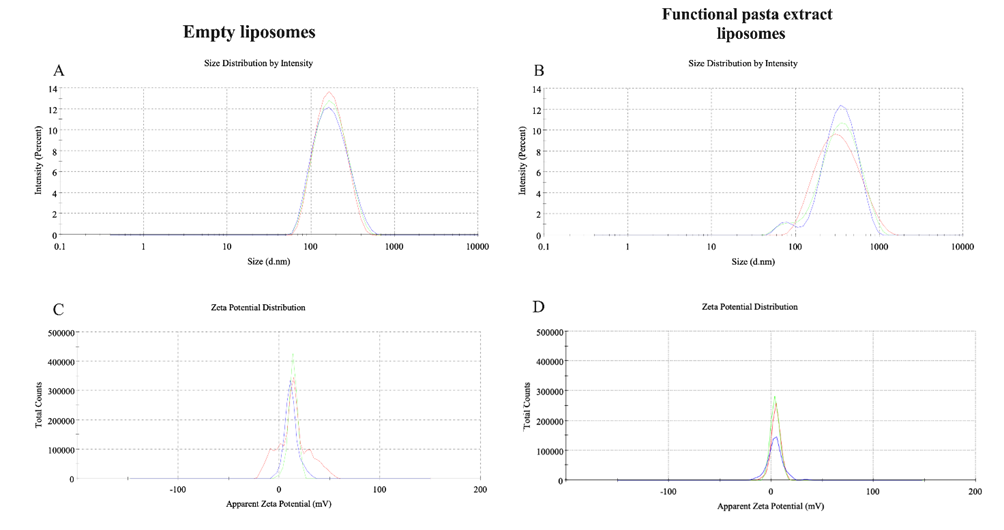


**Supplementary Figure 1.** *Physico-chemical characterization of liposomal suspensions performed by the Zetasizer NanoZS. Data evidenced an increase in vesicular size from empty liposome (panel A) to the FPE formulation (panel B), as well as a slight decrease in surface charge (panel C and D, respectively).*
