# Supplementary material for: Ancient grain flour consumption as a novel therapeutic approach for irritable bowel syndrome
Source: Eur J Nutr. 2025 Dec 19;65(1):11. doi: 10.1007/s00394-025-03859-8 (PMC12717202; doi:10.1007/s00394-025-03859-8)
Supplement: Supplementary file 2 — Supplementary Material 2 [file 394_2025_3859_MOESM2_ESM.doc]

**DIETARY RECOMMENDATIONS FOR IRRITABLE BOWEL SYNDROME (IBS)**

| **Supplementary table 1:** *Dietary recommendations for irritable bowel syndrome (IBS)* | | |
| --- | --- | --- |
| **CATEGORY** | **ALLOWED FOODS** | **NOT ALLOWED FOODS** |
| **CEREALS** | **Group 1:** New functional pasta (consumption frequency: 4 times per week), rice, polenta, oats, quinoa, tapioca, millet, amaranth, buckwheat, gluten-free bread and cereals, potatoes, potato flour. Rice, amaranth, and quinoa cakes. | Bread and baked goods, biscuits, croissants, wheat pasta (for **Group 1:** gluten-free pasta), wheat flour, spelt, kamut, barley, rye, couscous, semolina, lentil flour, muesli, breakfast cereals. |
|  | **Group 2:** Gluten-free pasta (consumption frequency: 4 times per week), rice, polenta, oats, quinoa, tapioca, corn, millet, amaranth, buckwheat, gluten-free bread and cereals, potatoes, potato flour. Rice/corn, amaranth, and quinoa cakes. |  |
| **DAIRY PRODUCTS** | Lactose-free milk, rice milk, oat milk, soy milk, and all plant-based beverages, lactose-free yogurt, soy yogurt, Greek yogurt, fruit sorbets. Aged hard cheeses. | Cow’s milk, goat’s milk, yogurt with lactose, fresh cheeses, ice cream, cream. |
| **NUTS** | Almonds, hazelnuts, walnuts, pine nuts. | Pistachios, cashews. |
| **VEGETABLES** | Carrots, pumpkin, Chinese cabbage, celery, lettuce, spinach, valerian, tomatoes, zucchini, eggplant, green beans, Swiss chard, chili pepper, aromatic herbs, olives, bamboo shoots, aromatic herbs. | Asparagus, cauliflower, garlic, onion, mushrooms, shallots, leeks, chicory, fennel, artichokes, Brussels sprouts, broccoli, radishes, bell peppers, turnips, Jerusalem artichokes. |
| **LEGUMES** | Peas. | Beans, chickpeas, lentils, soybeans. |
| **FRUITS** | Blueberries, strawberries, raspberries, melon, grapefruit, kiwi, oranges, lemons, limes, pineapple, passion fruit. Cooked chestnuts (max 10). | Apple, pear, watermelon, mango, apricot, avocado, cherries, peaches, plums, persimmons, lychee. Fruit juices. |
| **SWEETENERS** | White sugar, cane sugar, maple syrup, non-polyol sweeteners. | Agave, honey, fructose, xylitol, maltitol, mannitol, sorbitol, all sweeteners ending in “-ol” (chewing gum, candies). |

| **Supplememtary Table 2***: Primers for qRT-PCR analysis* | | |
| --- | --- | --- |
| Gene symbol | Forward (5′–3′) | Reverse (3′–5′) |
| *ALPI* | AGTTATCCTGCTCCCCACCTCCGG | GAAGGTCCAACGGCAGGACACCT |
| *SI* | TCCAGCTACTACTCGTGTGAC | CCCTCTGTTGGGAATTGTTCTG |
| *SLC16A1* | CACTTAAAATGCCACCAGCA | AGAGAAGCCGATGGAAATGA |
| *MS4A12* | TTGGAGTTCTTCGTAGCTTGTG | CAGGACAGACATATTGGTTGTG |
| *NLRP3* | AAGGGCCATGGACTATTTCC | GACTCCACCCGATGACAGTT |
| *IL1β* | ATGATGGCTTATTACAGTGGCAA | GTCGGAGATTCGTAGCTGGA |
| *IL18* | AAACTATTTGTCGCAGGAATAAAGAT | GCTTGCCAAAGTAATCTGATTCC |
| *GPX1* | CCC AAG CTC ATC ACC TGG TC | TGT CAA TGG TCT GGA AGC GG |
| *GPX4* | GAA ATG CCA TCA AGT GGA AC | CAG GTC CTT CTC TAT CAC CA |
| *SOD1* | GCAGATGACTTGGGCAAAGG | TGGGCGATCCCAATTACACC |
| *β-ACTIN* | TCCCTGGAGAAGAGCTACGA | AGGAAGGAAGGCTGGAAGAG |
| Abbreviations: *ALPI: Intestinal Alkaline Phosphatase; SI:* *Sucrase-Isomaltase; SLC16A1:* *Solute Carrier Family 16 Member 1; MS4A12: Membrane-Spanning 4-Domains Subfamily A Member 12; NLRP3:* *NLR Family Pyrin Domain Containing 3; IL1β:* *Interleukin 1 Beta; IL18: Interleukin 18; GPX1:* *Glutathione Peroxidase 1; GPX4: Glutathione Peroxidase 4; SOD1:* *Superoxide dismutase type 1.* | | |

| **Supplemental Table 3*.*** *Changes in clinical and instrumental parameters of participants divided by treatment group after 4 weeks (ITT analysis)* | | | |
| --- | --- | --- | --- |
| Variables | Control  (n=22) | Intervention  (n=20) | *p*-value |
| Δ Weight (kg) | 0.2±4 | -1.1±2 | 0.18 |
| Δ BMI (kg/m²) | -0.1±0.9 | -0.6±0.8 | 0.09 |
| Δ Abdominal pain severity (%) | -16±24 | -27±30 | 0.20 |
| Δ Abdominal bloating severity (%) | -20±29 | -37±29 | 0.07 |
| Δ Satisfaction with bowel habits (%) | -19±33 | -48±46 | 0.026 |
| Δ Straining during defecation | -0.2±0.5 | -0.4±0.9 | 0.45 |
| Δ Incomplete evacuation | -0.8±1 | -0.7±2 | 0.78 |
| Δ Painful defecation | -0.2±0.5 | -0.2±0.9 | 0.90 |
| Δ Hard stools | 0.1±1.4 | -0.8±1.4 | 0.05 |
| Δ Soft stools | -0.9±1 | -1.1±1 | 0.66 |
| Δ Fragmented defecation | -0.5±0.9 | -0.8±1 | 0.38 |
| Δ Urgency to defecate | -0.9±1 | -0.5±1 | 0.31 |
| Δ Fecal/gas incontinence | -0.1±0.6 | -0.5±1.1 | 0.20 |
| Δ Abdominal pain (days/week) | -0.5±1.2 | -1.5±1.2 | 0.015 |
| Δ Bloating (days/week) | -0.9±1.3 | -1.0±1.1 | 0.71 |
| Δ Laxative use (per week) | 0.2±1.0 | -0.1±0.4 | 0.18 |
| Δ Enemas and/or suppositories (per week) | 0.1±0.5 | 0.1±0.5 | 0.80 |
| Estimated satisfaction with treatment | 6±3 | 8±2 | 0.06 |
| Perceived improvement in symptomatology score | 2.7±1.5 | 2.1±1.7 | 0.28 |
| FARS score | 21±5 | 23±3 | 0.33 |
| Δ FS – 36 scale | 6.0±11.3 | 10.7±15.9 | 0.13 |
| Δ HADS-A | -2.1±3.9 | -1.9±4.3 | 0.50 |
| Δ HADS- D | -0.7±2.3 | -0.4±2.8 | 0.60 |
| Abbreviation: Δ =changes, BMI = Body Mass Index, FS-36=36-Item Short Form Survey, HADS-A= Hospital Anxiety and Depression Scale – Anxiety subscale A, HADS-D= Hospital Anxiety and Depression Scale – Depression subscale D, FARS score= Functional Assessment Rating Scale | | | |

| **Supplementary Table 4.** *Physical-chemical characterization of lipid vesicles* | | | |
| --- | --- | --- | --- |
| SAMPLE | SIZE (nm) | PDI | Z-POTENTIAL (mV) |
| Empty Liposomes | 159.3±1.179 | 0.170±0.009 | 13.2±1.10 |
| FPE Liposomes | 280.2±11.07 | 0.243±0.004 | 4.23±0.355 |
| Abbreviation: FPE, Functional Pasta Extract; PDI, Poly Dispersion Index | | | |
