# Supplementary material for: Ancient grain flour consumption as a novel therapeutic approach for irritable bowel syndrome
Source: Eur J Nutr. 2025 Dec 19;65(1):11. doi: 10.1007/s00394-025-03859-8 (PMC12717202; doi:10.1007/s00394-025-03859-8)
Supplement: Supplementary file 4 — Supplementary Material 4 [file 394_2025_3859_MOESM4_ESM.pdf]

Figure 5D – p-ERK 1/2 42-44 kDa

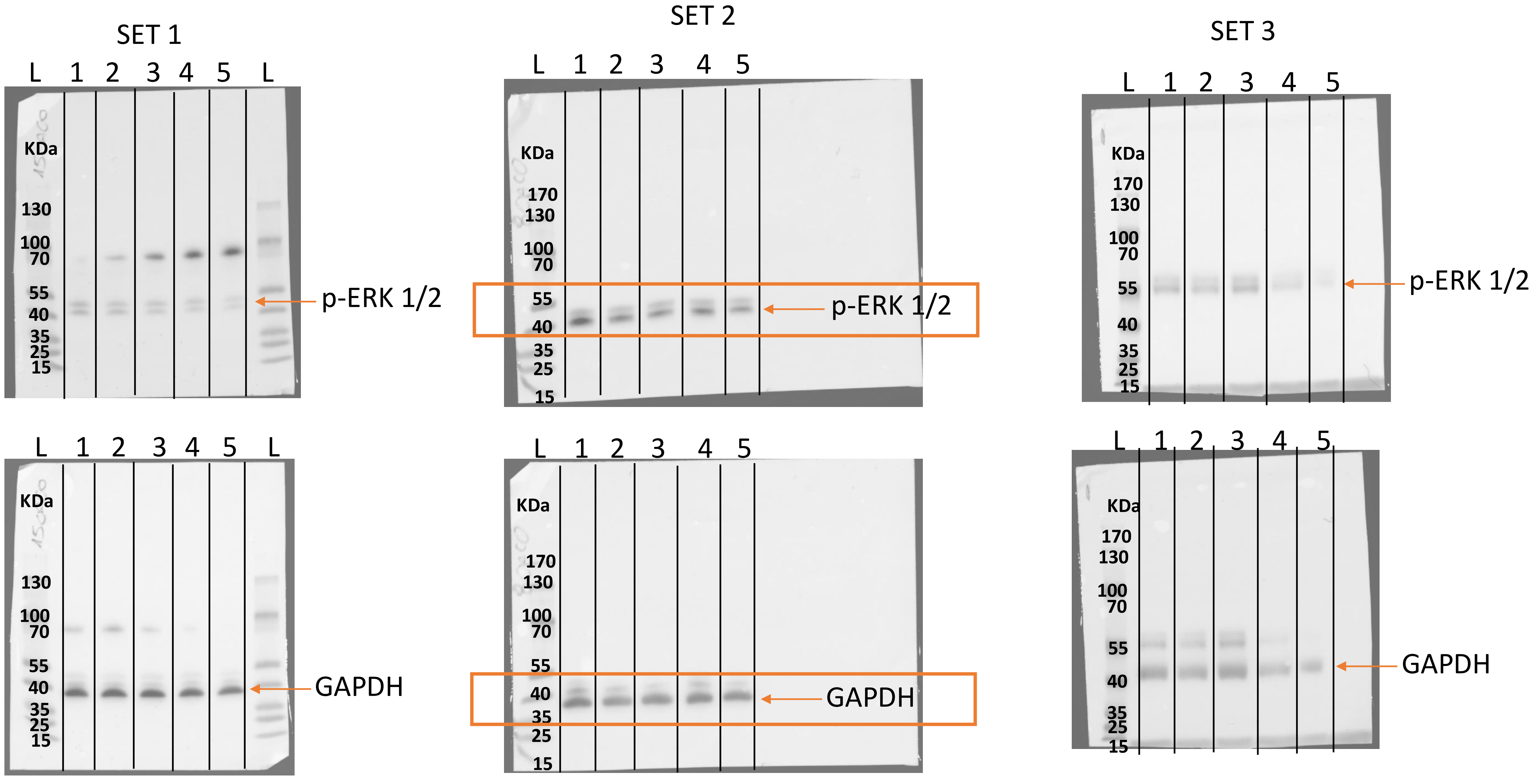

L: ladder; 1: Untreated; 2: TNFα 20 ng/mL ; 3: TNFα 20 ng/mL + FPE 0.25 μg/mL ; 4: TNFα 20 ng/mL + FPE 0.5 μg/mL ; 5: TNFα 20 ng/mL + FPE 1 μg/mL

Figure 5D – NF-kB 65 kDa

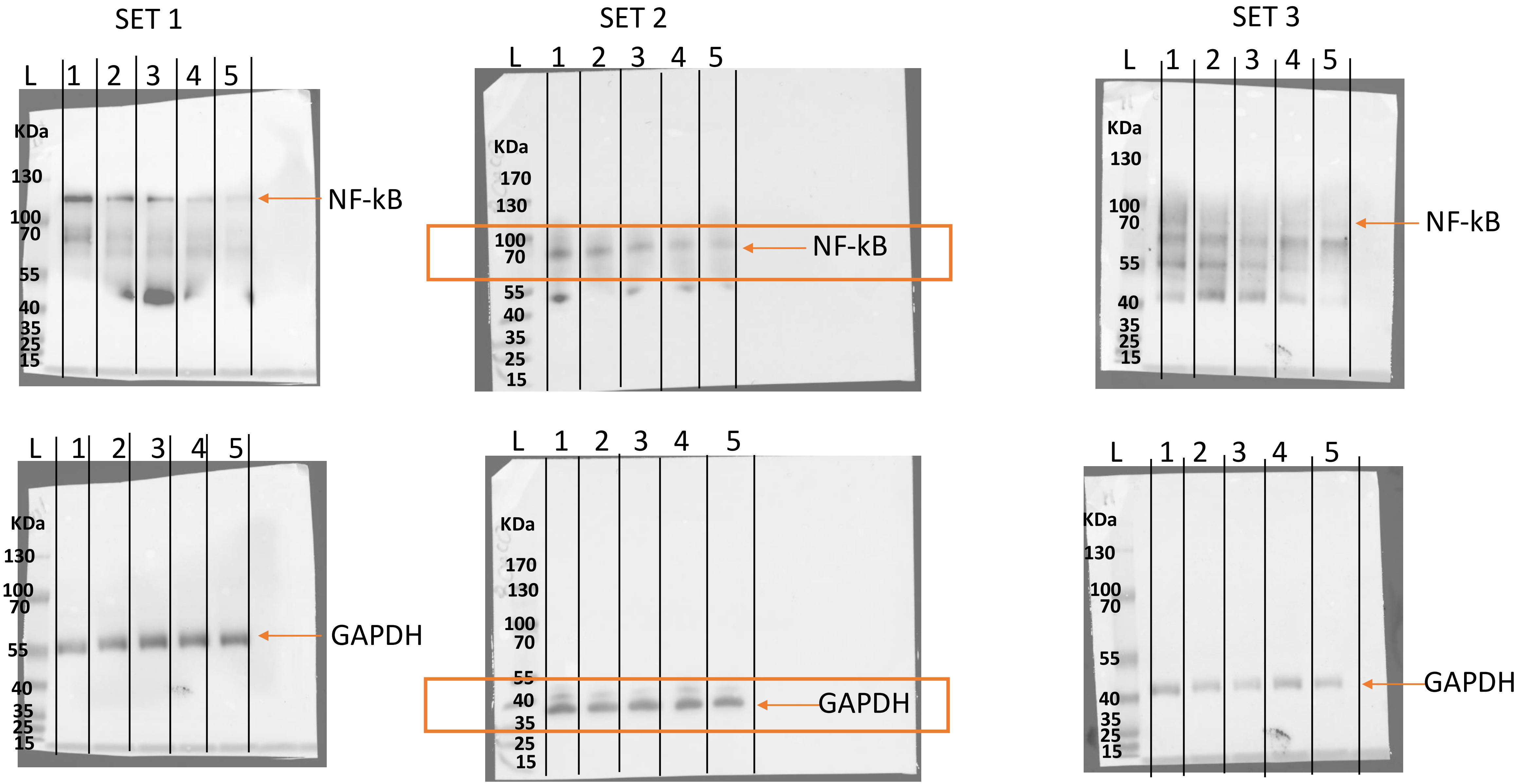

L: ladder; 1: Untreated; 2: TNFα 20 ng/mL ; 3: TNFα 20 ng/mL + FPE 0.25 µg/mL ; 4: TNFα 20 ng/mL + FPE 0.5 µg/mL ; 5: TNFα 20 ng/mL + FPE 1 µg/mL

Figure 5D – p-ERK 1/2 42-44 kDa

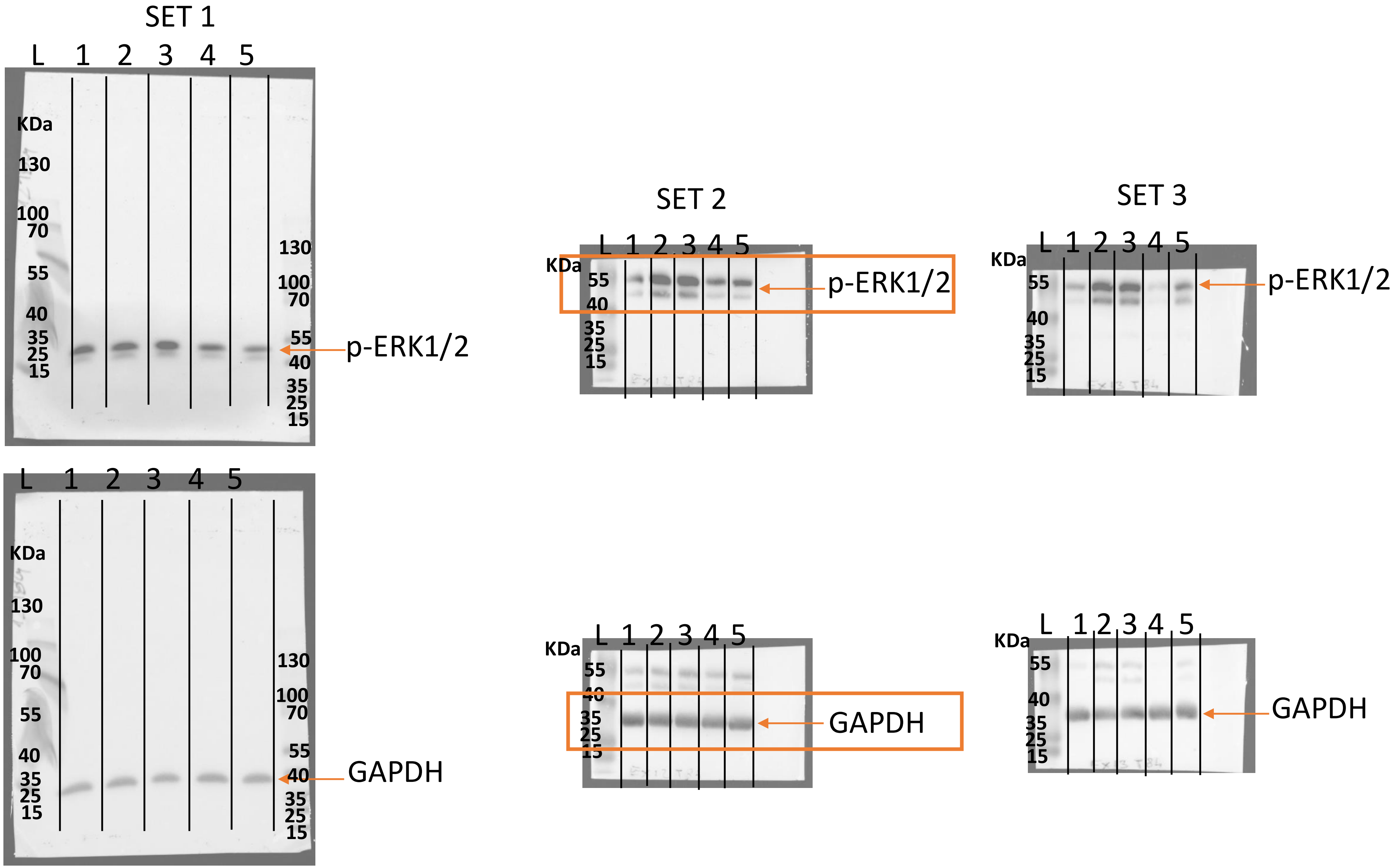

L: ladder; 1: Untreated; 2: TNFα 20 ng/mL ; 3: TNFα 20 ng/mL + FPE 0.25 µg/mL ; 4: TNFα 20 ng/mL + FPE 0.5 µg/mL ; 5: TNFα 20 ng/mL + FPE 1 µg/mL

Figure 6D – NF-kB 65 kDa

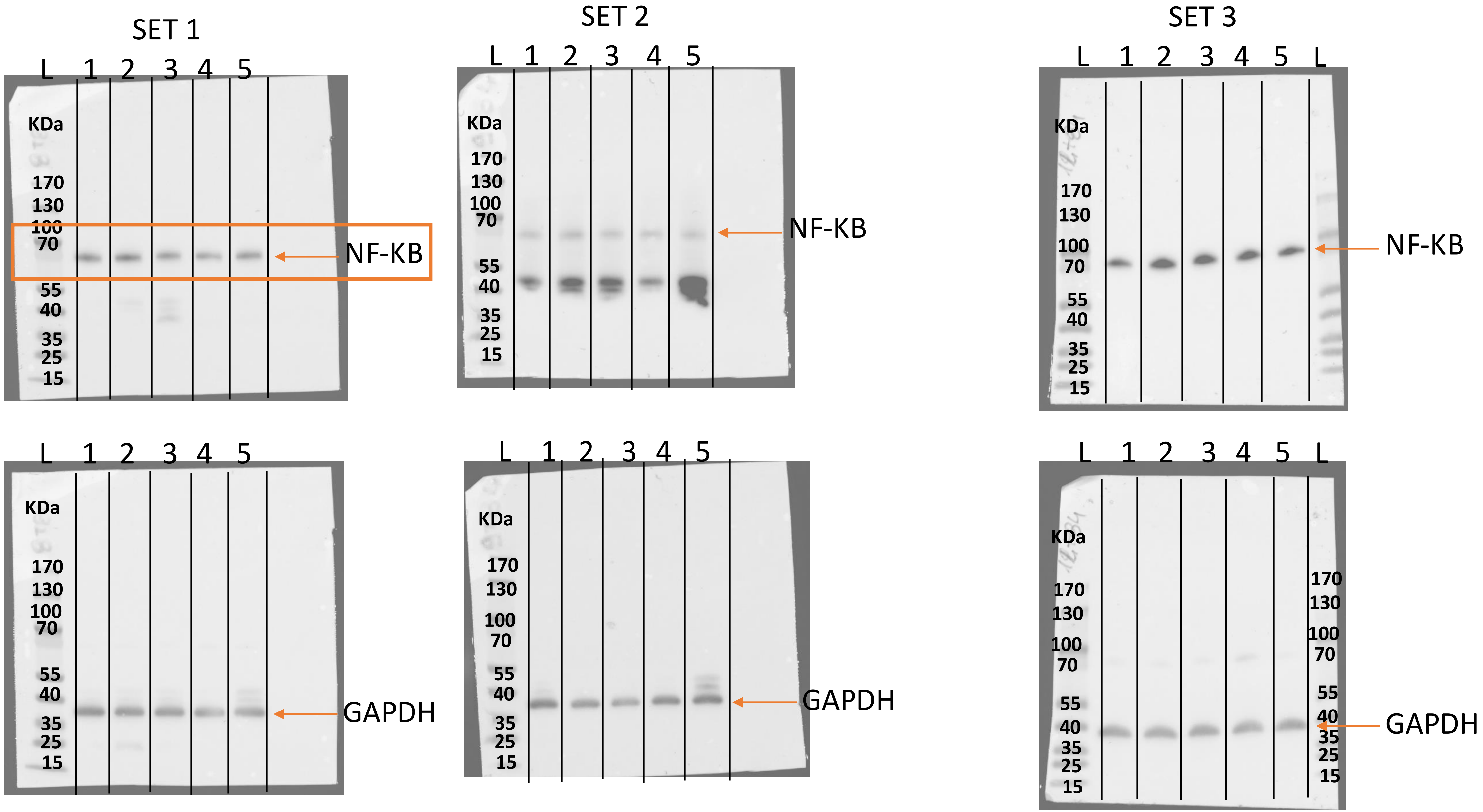

L: ladder; 1: Untreated; 2: TNFα 20 ng/mL ; 3: TNFα 20 ng/mL + FPE 0.25 µg/mL ; 4: TNFα 20 ng/mL + FPE 0.5 µg/mL ; 5: TNFα 20 ng/mL + FPE 1 µg/mL
